# Systems Biology of Aromatic Compound Catabolism in Facultative Anaerobic *Aromatoleum aromaticum* EbN1^T^

**DOI:** 10.1128/msystems.00685-22

**Published:** 2022-11-29

**Authors:** Patrick Becker, Sarah Kirstein, Daniel Wünsch, Julia Koblitz, Ramona Buschen, Lars Wöhlbrand, Boyke Bunk, Christina Hinrichs, Sabine Kaltenhäuser, Jochen Lehmann, Gesa Martens, Britta Katrin Ripken, S. Alexander Riemer, Philipp Stoltenberg, Daniela Thies, Kathleen Trautwein, Esther V. Wenzel, Ida Schomburg, Michael Winklhofer, Dietmar Schomburg, Meina Neumann-Schaal, Ralf Rabus

**Affiliations:** a General and Molecular Microbiology, Institute for Chemistry and Biology of the Marine Environment (ICBM), Carl von Ossietzky University of Oldenburg, Oldenburg, Germany; b Department of Bioinformatics and Biochemistry, Institute for Biochemistry, Biotechnology and Bioinformatics, Technische Universität Carolo-Wilhelmina zu Braunschweig, Braunschweig, Germany; c Braunschweig Integrated Centre of Systems Biology (BRICS), Technische Universität Carolo-Wilhelmina zu Braunschweig, Braunschweig, Germany; d Leibniz Institute DSMZ-German Collection of Microorganisms and Cell Cultures GmbH, Braunschweig, Germany; e Department of Microbiology, Max Planck Institute for Marine Microbiology, Bremen, Germany; f Research Center Neurosensory Science, Carl von Ossietzky University of Oldenburg, Oldenburg, Germany; g Sensory Biology of Animals, Institute of Biology and Environmental Sciences (IBU), Carl von Ossietzky University of Oldenburg, Oldenburg, Germany; University of Waterloo

**Keywords:** systems biology, multi-omics, metabolic network, metabolic model, growth physiology, aromatic compounds, facultative anaerobe, *Aromatoleum aromaticum* EbN1^T^

## Abstract

Members of the genus *Aromatoleum* thrive in diverse habitats and use a broad range of recalcitrant organic molecules coupled to denitrification or O_2_ respiration. To gain a holistic understanding of the model organism *A. aromaticum* EbN1^T^, we studied its catabolic network dynamics in response to 3-(4-hydroxyphenyl)propanoate, phenylalanine, 3-hydroxybenzoate, benzoate, and acetate utilized under nitrate-reducing versus oxic conditions. Integrated multi-omics (transcriptome, proteome, and metabolome) covered most of the catabolic network (199 genes) and allowed for the refining of knowledge of the degradation modules studied. Their substrate-dependent regulation showed differing degrees of specificity, ranging from high with 3-(4-hydroxyphenyl)propanoate to mostly relaxed with benzoate. For benzoate, the transcript and protein formation were essentially constitutive, contrasted by that of anoxia-specific versus oxia-specific metabolite profiles. The matrix factorization of transcriptomic data revealed that the anaerobic modules accounted for most of the variance across the degradation network. The respiration network appeared to be constitutive, both on the transcript and protein levels, except for nitrate reductase (with *narGHI* expression occurring only under nitrate-reducing conditions). The anoxia/nitrate-dependent transcription of denitrification genes is apparently controlled by three FNR-type regulators as well as by NarXL (all constitutively formed). The resequencing and functional reannotation of the genome fostered a genome-scale metabolic model, which is comprised of 655 enzyme-catalyzed reactions and 731 distinct metabolites. The model predictions for growth rates and biomass yields agreed well with experimental stoichiometric data, except for 3-(4-hydroxyphenyl)propanoate, with which 4-hydroxybenzoate was exported. Taken together, the combination of multi-omics, growth physiology, and a metabolic model advanced our knowledge of an environmentally relevant microorganism that differs significantly from other bacterial model strains.

**IMPORTANCE** Aromatic compounds are abundant constituents not only of natural organic matter but also of bulk industrial chemicals and fuel components of environmental concern. Considering the widespread occurrence of redox gradients in the biosphere, facultative anaerobic degradation specialists can be assumed to play a prominent role in the natural mineralization of organic matter and in bioremediation at contaminated sites. Surprisingly, differential multi-omics profiling of the *A. aromaticum* EbN1^T^ studied here revealed relaxed regulatory stringency across its four main physiological *modi operandi* (i.e., O_2_-independent and O_2_-dependent degradation reactions versus denitrification and O_2_ respiration). Combining multi-omics analyses with a genome-scale metabolic model aligned with measured growth performances establishes *A. aromaticum* EbN1^T^ as a systems-biology model organism and provides unprecedented insights into how this bacterium functions on a holistic level. Moreover, this experimental platform invites future studies on eco-systems and synthetic biology of the environmentally relevant betaproteobacterial *Aromatoleum*/*Azoarcus*/*Thauera* cluster.

## INTRODUCTION

Aromatic compounds occur in the biosphere as constituents of major biomacromolecules (lignin, proteins) (1), as phytohormones (2), or as conversion products in anoxic aquatic sediments (3). Moreover, they are encountered in large quantities in the geosphere as prominent constituents of insoluble macromolecular organic matter (kerogen) (4) and crude oil (5), from which they can enter the biosphere via geological processes and anthropogenic activities. Finally, aromatic compounds are widely used in large quantities during industrial synthesis (6). Taken together, the structurally diverse aromatic compounds represent, next to carbohydrates, the second most abundant class of organic molecules on the planet. Aromatic compounds are Janus-faced substrates for microorganisms; their energy richness makes them attractive, but, in contrast, their chemical stability is demanding (7), and their toxicology is problematic (6, 8). To meet the biochemical challenge involved in the activation and ring cleavage of aromatic compounds, aerobic microorganisms employ O_2_-dependent oxygenase-based catalysis (9), whereas anaerobes have evolved a variety of O_2_-independent reactions that suit these purposes (e.g., [10, 11]).

Many habitats of microorganisms are characterized by redox gradients, meaning transitions between oxic and anoxic conditions. In accordance with this, appropriately adapted aromatic compound-degrading bacteria have been isolated and studied. These include the genome-sequenced aerobic degradation specialists of *Burkholderiaceae* (e.g., Burkholderia xenovorans) (12), and *Pseudomonadaceae* (e.g., Pseudomonas putida) (13), versus the strictly anaerobic ones of iron-reducing *Geobacteraceae* (e.g., Geobacter metallireducens GS-15) (14), and sulfate-reducing *Desulfobacteraceae* (e.g., Desulfobacula toluolica Tol2) (15). An intermediary position is assumed by the facultative denitrifying degradation specialists of the betaproteobacterial genera *Aromatoleum* (16) and *Thauera* (17). To date, the genus *Aromatoleum* accommodates a dozen taxonomically described denitrifying species that anaerobically degrade a large variety of aromatic compounds. Among *Aromatoleum* species, Aromatoleum aromaticum EbN1^T^ is particularly well-studied on the physiological, proteogenomic, and metabolite level (e.g., [18–22]) and was previously proposed as a promising model for systems biology-level investigations (23).

To better understand a microorganism's adaptability, environmental role, and services in its natural habitat, holistic perspectives on its metabolism are required, as are provided by systems biology (e.g., [24–27]). Systems biology is based on two main approaches. First, multi-omics data are integrated to unravel the global physiological response of a given study organism to each test condition and to lay the foundation for metabolic network construction. Second, genome-scale models of the reconstructed metabolism are aligned with the experimentally determined physiological performance to construct a framework for a predictive understanding of microorganisms. Ultimately, a systems-biology level understanding of environmentally relevant bacteria will form a valuable basis for eco-systems biology (28).

**FIG 1:**
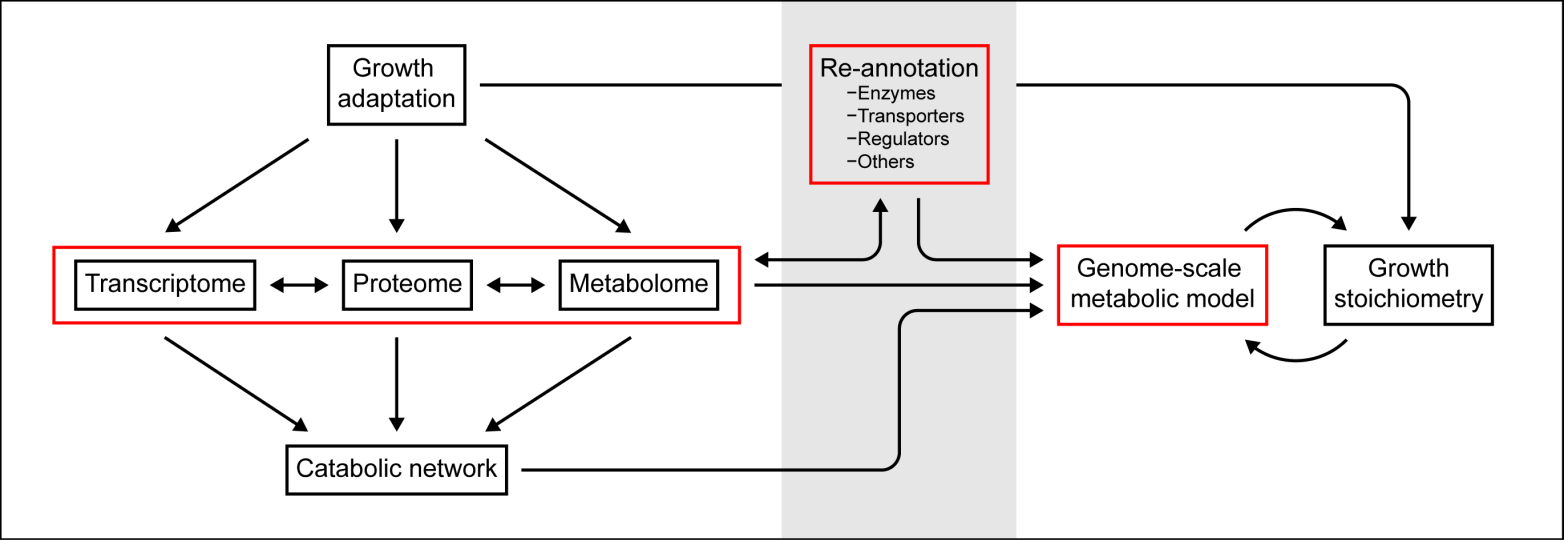
Experimental design of the present study. Multi-omics approaches and manual genome reannotation were integrated with a genome-scale metabolic model and quantitative growth stoichiometry.

The overarching motivation of this study was to establish *A. aromaticum* EbN1^T^ as a model organism for systems biology-level investigations of facultative anaerobic degradation specialists, which could potentially be expanded to other members of the *Aromatoleum*/*Azoarcus*/*Thauera* cluster. For this purpose, the following two aims were pursued: (i) the application of differential multi-omics to elucidate the dynamics of the degradation and respiration networks in response to selected aromatic substrates on which *A. aromaticum* EbN1^T^ can thrive, under both anoxic (nitrate-reducing) and oxic conditions; (ii) the development of a genome-scale metabolic model on the basis of the manually reannotated genome of *A. aromaticum* EbN1^T^ and the comparison of it to quantitative growth stoichiometric data. The integration of the approaches is shown schematically in Fig. 1.

## RESULTS AND DISCUSSION

### Catabolic network.

With *A. aromaticum* EbN1^T^ as a facultative anaerobic degradation specialist at the focal point of this study, four aromatic growth substrates (plus acetate as a control) that are degraded via reaction sequences that are assumed to operate exclusively under anoxic conditions, exclusively under oxic conditions, or both were selected. These 10 different growth conditions (for the workflow and the reproducibility of the cultivation, see Fig. S1A and B) were profiled globally on the transcript, protein, and metabolite levels. Genome-based predictions were integrated with the differential multi-omics data to construct the degradation and respiration networks and to investigate their growth condition-dependent regulation.

**FIG 2:**
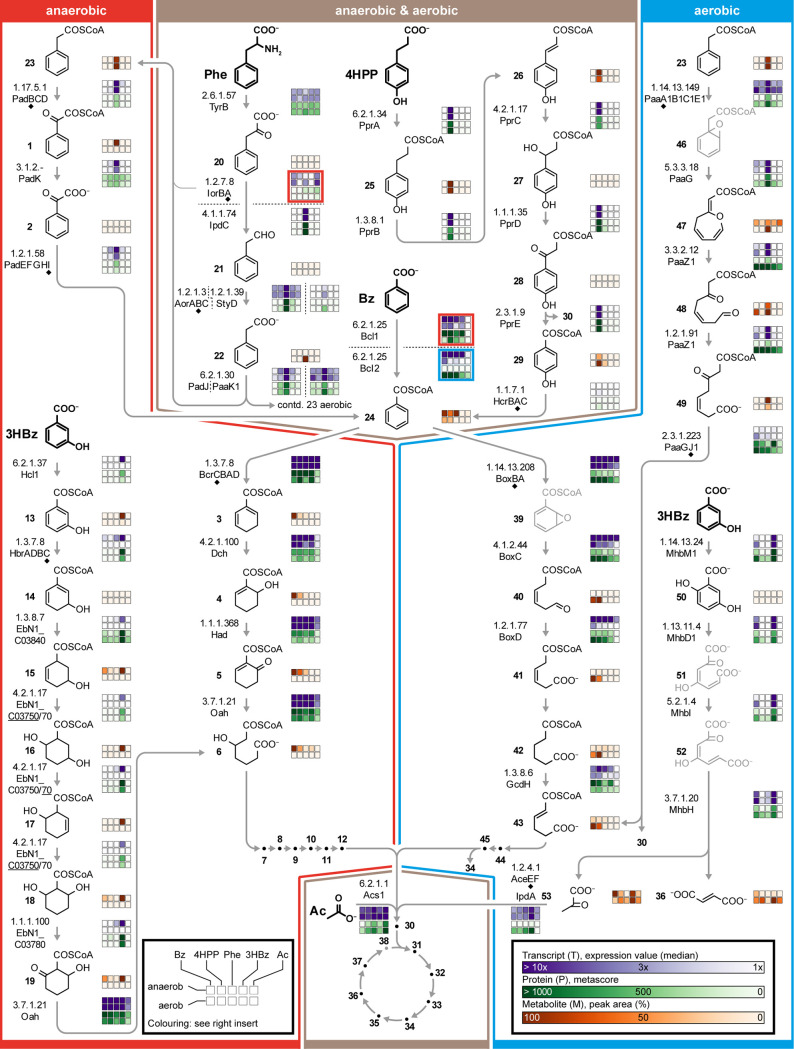
Degradation network of *A. aromaticum* EbN1^T^ integrating substrate-specific profiles of transcripts, proteins, and metabolites. The three major sections of this network (i.e., anaerobic, anaerobic & aerobic, and aerobic) are marked by red, brown, and blue boxes, respectively. Abbreviations of adaptation substrates (marked in bold, alphabetic order): Ac, acetate; Bz, benzoate; 3HBz, 3-hydroxybenzoate; 4HPP, 3-(4-hydroxyphenyl)propanoate; Phe, phenylalanine. Compound names of degradation intermediates are given in Table S2. In cases of multimeric enzymes, the catalytically active subunit (marked by a filled diamond) was selected for displaying transcript and protein profiles. In case a specific reaction can be catalyzed by alternative enzymes, transcript and protein abundances are displayed for the underlined one. Underlying transcriptomic and proteomic data are provided in Table S1, and data on the identification and abundance profiles of metabolites in are provided in Table S2. The gray coloring of structural formulae indicates that the metabolite was not identified. The differential multi-omics data of the TCA cycle are schematically shown in Fig. S2, and the underlying data are shown in Table S1.

**(i) Structure of the degradation network.** The network for the anaerobic as well as aerobic degradation of phenylalanine, 3-(4-hydroxyphenyl)propanoate, 3-hydroxybenzoate, and benzoate to CO_2_ is illustrated in Fig. 2. The abundance profiles of the detected transcripts, proteins, and metabolites are compiled in Tables S1 and S2. This degradation network is composed of 60 enzymatic reactions, 53 metabolites (41 were detected), and 108 transcripts/proteins (101 transcripts/96 proteins were detected), which were covered overall by 77.4 to 93.5% on the omics level. The degradation network can be subdivided into three major sections (only anaerobic, anaerobic & aerobic, and only aerobic), which are briefly presented in the following.

10.1128/msystems.00685-22.6 TABLE S1 Transcriptomic and proteomic data (degradation network, transporters, sensory/regulatory systems, metabolic model components, PHB formation). Download Table S1, XLSX file, 0.2 MB. Copyright © 2022 Becker et al. 2022 Becker et al. https://creativecommons.org/licenses/by/4.0/ This content is distributed under the terms of the Creative Commons Attribution 4.0 International license.

10.1128/msystems.00685-22.7 TABLE S2 Metabolomic data (degradation network, small metabolites, CoA esters). Download Table S2, XLSX file, 0.05 MB. Copyright © 2022 Becker et al. 2022 Becker et al. https://creativecommons.org/licenses/by/4.0/ This content is distributed under the terms of the Creative Commons Attribution 4.0 International license.

10.1128/msystems.00685-22.2 FIG S2 Multi-omics of the TCA cycle. Download FIG S2, PDF file, 0.6 MB. Copyright © 2022 Becker et al. 2022 Becker et al. https://creativecommons.org/licenses/by/4.0/ This content is distributed under the terms of the Creative Commons Attribution 4.0 International license.

Specific anaerobic pathways are used for phenylacetyl-CoA (derived from phenylalanine), 3-hydroxybenzoate, and benzoyl-CoA (derived from 3-(4-hydroxyphenyl)propanoate and benzoate, respectively). The conversion of phenylacetyl-CoA to benzoyl-CoA involves the formation of phenylglyoxylyl-CoA and its further hydrolysis to phenylglyoxylate, followed by its decarboxylation (e.g., [29]). The anaerobic degradation of 3-hydoxybenzoate involves reductive dearomatization, forming 3-hydroxydienoyl-CoA followed by a modified β-oxidation yielding 3-hydroxypimeloyl-CoA (e.g., [19]). Similarly, benzoate is degraded via dienoyl-CoA and 3-hydroxypimeloyl-CoA, according to the central anaerobic benzoyl-CoA pathway (e.g., [30]).

Pathways/reactions shared in anaerobic as well as aerobic degradation comprise the transformation of phenylalanine to phenylacetyl-CoA (including deamination and decarboxylation) (e.g., [31]) and of 3-(4-hydroxyphenyl)propanoate to benzoyl-CoA (including β-oxidation and presumptive reductive dehydroxylation) (e.g., [22]), as well as the activation of benzoate to its CoA-ester. Phenylalanine degradation is multifaceted, as the intermediate phenylpyruvate can also be directly converted to phenylacetyl-CoA by anaerobic indolpyruvate:ferredoxin oxidoreductase (IorAB) (19) and phenylacetaldehyde can be oxidized to phenylacetate by AorABC (anaerobic & aerobic) (32) or by StyD (aerobic, synonym PDH) (33). For the activation of phenylacetate and benzoate to the respective CoA-esters, dedicated CoA-ligases are used under anoxic and/or oxic conditions (e.g., [34, 35]). The further degradation of the intermediates phenylacetyl-CoA and benzoyl-CoA proceeds via specific anaerobic and aerobic routes, resulting in hybrid pathways.

The aerobic section of the network includes several conspicuous reactions. Phenylacetyl-CoA is degraded via the key metabolites 2-(1,2-epoxy-1,2-dihydrophenyl)acetyl-CoA and 2-oxepin-2[3H]-ylideneacetyl-CoA to ultimately branch into the aerobic “box” pathway on the level of *trans*-2,3-didehydroadipyl-CoA (e.g., [36]). The activation of 3-hydroxybenzoate by a monooxygenase feeds into the gentisate pathway, producing pyruvate and fumarate (e.g., [37]). Benzoyl-CoA is degraded via the “box pathway” involving the key intermediate 2-(1,2-epoxy-1,2-dihydrophenyl)acetyl-CoA (e.g., [38]).

All of the routes of the degradation network eventually yield acetyl-CoA, fumarate, or succinyl-CoA, which are completely oxidized to CO_2_ via the canonical TCA cycle.

According to chromosomal colocalization and differential expression profiles, the uptake of the aromatic substrates is mostly mediated by ATP-energized ABC-type transporters (e.g., in the case of phenylalanine and 3-(4-hydroxyphenyl)propanoate). Nonetheless, H^+^-gradient-driven permeases are also involved (e.g., TRAP- and MFS-type systems for 3-hydroxybenzoate under anoxic and oxic conditions, respectively). More details are provided in Table S1.

**(ii) Regulatory dynamics across the degradation network.** The abundance profiles of individual transcripts, proteins, and metabolites across the 10 growth conditions studied are superimposed onto the degradation network (Fig. 2). The observed profiles ranged from highly specific to various degrees of relaxation, as exemplified in the following. During anaerobic or aerobic growth with 3-(4-hydroxyphenyl)propanoate, transcripts, proteins, and metabolites for all consecutive reaction steps to the level of 4-hydroxybenzoyl-CoA were formed at a high abundance and with a high substrate specificity (i.e., they were not detectable or were only detected at negligible abundances under the other eight growth conditions). Similarly, the pathway for the anaerobic degradation of 3-hydroxybenzoate was specifically regulated, with some exceptions on the transcript level (e.g., *EbN1_C03750/70*). Distinctively less-specific abundance patterns were observed in the case of benzoate degradation. A benzoate CoA-ligase (Bcl1 versus Bcl2) is encoded in close proximity to each gene cluster for anaerobic and aerobic benzoate degradation, suggesting corresponding functional assignments. However, *bcl1* and *bcl2* were both expressed during anaerobic as well as aerobic growth, as was also observed for the two phenylacetate CoA-ligase encoding genes (*padJ* and *paak1*). Metabolites of the anaerobic benzoyl-CoA pathway (e.g., dienoyl-CoA) and of the aerobic “box” pathway (e.g., 3,4-didehydroadipyl-CoA semialdehyde) were specifically detected under the respective electron acceptor conditions. Surprisingly, however, transcripts and proteins assigned to the two benzoate degradation pathways were formed under essentially all of the tested growth conditions, suggesting a regulatory uncoupling between protein formation and activity. As expected, the transcripts and proteins of the TCA cycle were formed with high abundances across essentially all of the growth conditions (Fig. S2).

Chromosomal colocalization indicated several candidates for regulators controlling the gene expression of the studied degradation pathways, as outlined in the following. The *EbN1_C30520-70* gene cluster for the degradation of 3-(4-hydroxyphenyl)propanoate is under the control of a TetR-like transcriptional repressor (EbN1_C30510, renamed PprR) in conjunction with catabolite repression (by benzoate) and another yet unknown transcriptional activation mechanism, as previously reported (22, 39). The findings for the transcriptional regulation of the anaerobic versus aerobic degradation pathways for phenylacetyl-CoA (phenylalanine-derived) were more complex, with the genes for all three of the assigned regulators being expressed under both respiratory conditions: *padR* (*EbN1_C30890*) being associated with the anaerobic pathway (*pad* gene cluster), as well as *EbN1_C32850/60* (two-component system) for the aerobic pathway (1^st^ *paa* gene cluster, and the products are a part of the degradation network in Fig. 2) and *EbN1_C20230* (TetR-family member, encoded next to the paralogous 2^nd^ *paa* gene cluster, no products formed). A schematic overview of the sensory/regulatory proteins assigned to the degradation modules, including genetic localization, is presented in Fig. S3A–H (for further details, see Table S1).

10.1128/msystems.00685-22.3 FIG S3 Sensory/regulatory systems related to degradation and respiration networks. Download FIG S3, PDF file, 0.8 MB. Copyright © 2022 Becker et al. 2022 Becker et al. https://creativecommons.org/licenses/by/4.0/ This content is distributed under the terms of the Creative Commons Attribution 4.0 International license.

**FIG 3:**
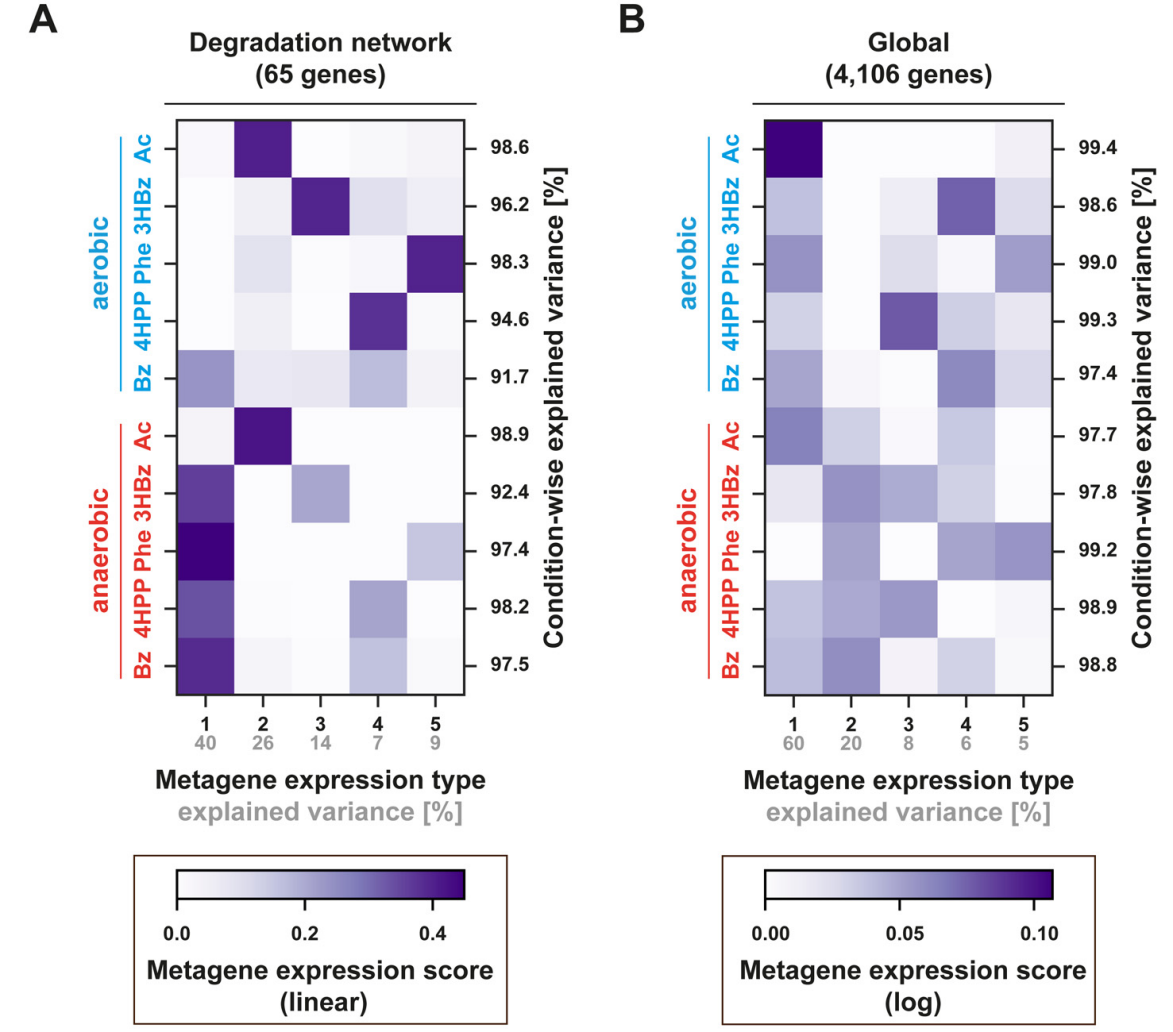
Commonalities and differences in gene expression patterns across the 10 growth conditions as revealed by the nonnegative matrix factorization of the transcript matrix into five metagene expression types (“components”) and scores (“contributions”). (A) Focus on the specific genes of the degradation network (Fig. 2), where only one transcript sequence is considered per reaction (except for β-oxidation), which, in the case of multimeric enzymes, represents the transcript for the catalytic subunit, totaling 65 distinct genes. Here, the first metagene expression type (explaining 40% of the total variance) is due largely to the anaerobic degradation of aromatic compounds. Together, the five components explain 95% of the total variance. The actual metagene expression types and details on the partitioning of the cumulative explained variance are provided in Fig. S4A. Note that in order to emphasize the main differences between a limited number of genes with marked expression profiles, we here analyzed transcripts on a linear scale. For comparison, we also provide a similar analysis for log-scaled transcripts in Fig. S4C and D. (B) Global view of the transcriptome data set of all chromosomal genes, revealing a high level of congruency in gene expression among oxic growth conditions (first metagene expression type, explaining 60% of the total variance), and a second component (explaining an additional 20% of the total variance) for anoxic growth conditions, followed by several minor metagene expression types for specific substrates, which by and large confirm the results obtained from the focus on the degradation network. Note that logarithmic scaling was applied to prevent highly abundant transcripts from dominating those with intermediate abundance. Together, the five metagene expression types explain 99% of the total variance. Further details are provided in Fig. S4B. The abbreviations of the adaptation substrates are as described in the legend of Fig. 2.

To identify similarities and differences in the gene-expression profiles among the 10 studied growth conditions, we analyzed the transcriptome data set via nonnegative matrix factorization (NMF), which decomposes a high-dimensional data matrix into positive linear combinations of a few end-member components, which are referred to as metagene expression types (40–42). Thus, NMF finds common sources of variation in gene expression across various growth conditions and thereby also reduces the dimensionality of the original data set without the loss of essential information. We first focused on the degradation network (Fig. 2), which is based on the 65 genes encoding the catalytic enzyme subunits. The first metagene expression type, which explains 40% of the network variance (Fig. 3A and S4A), is largely composed of the degradation modules of aromatic compounds with anoxia as the dominant regulatory determinant. The second metagene expression type represents both the anaerobic and the aerobic utilization of acetate. The degradation modules for the aerobic degradation of 3-(4-hydroxyphenyl)propanoate, phenylalanine, and 3-hydroxybenzoate are each factored into a specific metagene expression type (third, fourth, and fifth, respectively). In a second step, NMF was used to analyze the global transcriptome data set (Fig. 3B; Fig. S4B), in which the transcript counts were log-transformed, to focus on the broad body of genes with intermediate expression levels rather than on the few highly expressed ones, which would otherwise dominate the variance of the untransformed data set. In this global perspective, the first metagene expression type already accounted for 60% of the total variance, thus indicating a high degree of coherency in global gene expression (except for phenylalanine, anaerobic). The second confirms the aforementioned differences in regulatory effectiveness of anoxia versus oxia, whereas the third to fifth reflect substrate specificities fairly well. Taken together, the total transcriptional variance of the degradation network versus that of the global transcriptome can be explained by 96% versus 99%, each with just five metagene expression types.

10.1128/msystems.00685-22.4 FIG S4 Metagene expression analysis of the degradation network versus global transcriptomic data sets. Download FIG S4, PDF file, 0.9 MB. Copyright © 2022 Becker et al. 2022 Becker et al. https://creativecommons.org/licenses/by/4.0/ This content is distributed under the terms of the Creative Commons Attribution 4.0 International license.

**FIG 4:**
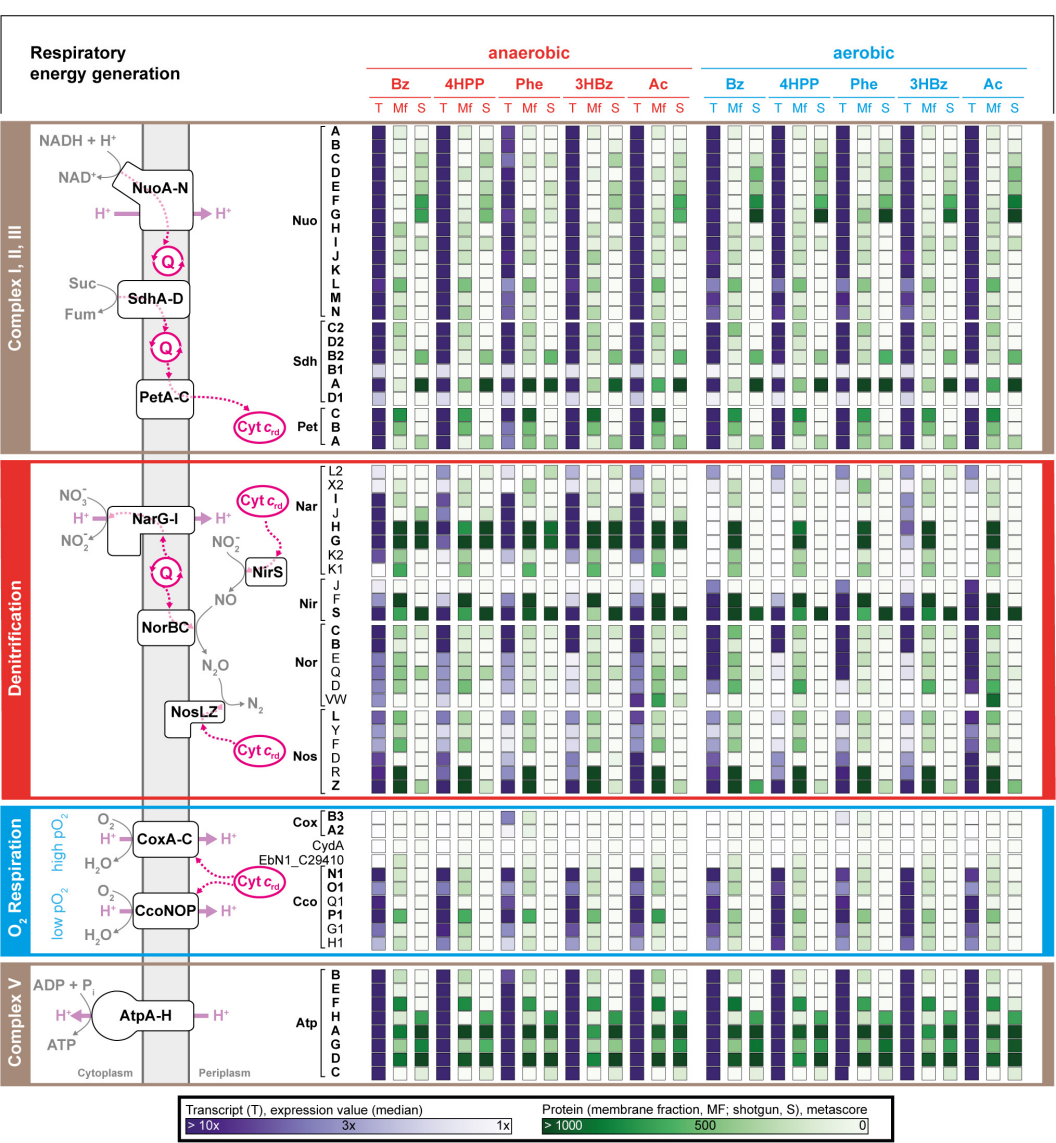
Respiration network of *A. aromaticum* EbN1^T^ integrating, substrate-specific profiles of transcripts and proteins. The color coding of the main network sections is as in Fig. 2. The abbreviations of the adaptation substrates are as described in the legend of Fig. 2. The underlying data are provided in Table S1.

**(iii) Respiration network.** The modes of respiratory energy conservation (denitrification versus O_2_ respiration) of *A. aromaticum* EbN1^T^, coupled to the above-described degradation network, as well as the respective growth condition-specific transcript and protein profiles are schematically integrated in Fig. 4 (for further details, see Table S1). This respiration network is composed of four major modules, essentially as is known from other proteobacteria, such as Pseudomonas spp. (43) and Escherichia coli (44). First, complexes I, II, and III conduct vectorial electron transport from NADH and succinate to periplasmic cytochrome *c*. The shared use of these complexes by the two respiratory modes is reflected in the consistent expression/synthesis of all subunits under all investigated conditions. Second, the denitrification module consists of nitrate (NO_3–_) reductase (NarGHI), nitrite (NO_2–_) reductase (NirS), nitric oxide (N_2_O) reductase (NorBC), and nitrous oxide (NO) reductase (NosZ), which together perform the dissimilatory reduction of NO_3–_ to N_2_. Although the *narGHI* genes were only expressed under nitrate-reducing conditions, surprisingly, their protein products were also detected under oxic conditions. All of the other genes/proteins of the denitrification module were expressed/formed under all tested conditions, with NirS and NorC displaying the highest abundances. Third, for O_2_ reduction, a low-affinity system (CcoNOP) and a high-affinity system (CoxABC) are encoded in the genome of *A. aromaticum* EbN1^T^. Notably, the Cox system was essentially not expressed/formed under any of the studied conditions, while the contrary was observed for the Cco system. Fourth, as expected, the transcripts and proteins of the ATP synthase (AtpA-H) were observed at high levels under all tested growth conditions.

**(iv) Superior O_2_/NO_3–_-discriminating regulation.** It is notable that the genome of *A. aromaticum* EbN1^T^ does not encode the global ArcBA regulatory system, which responds to the redox state and activates gene expression for aerobic metabolism (45). However, *A. aromaticum* EbN1^T^ is endowed with the global regulator FNR (fumarate nitrate reduction), which, from studies with E. coli K-12, is known to control the metabolic response to transitions from oxic to anoxic conditions (46). Dimeric FNR directly responds to O_2_ by converting its cubane [4Fe-4S]^2+^ clusters into planar [2Fe-2S]^2+^ clusters, thereby switching the regulator from its transcriptionally active form to its inactive form. The active dimer binds with conserved E–SR in its DNA-binding helix-turn-helix domain to the palindromic recognition motif TTGAT–N_4_–ATCAA and to variants thereof in the upstream regions of FNR-controlled promoters (47, 48). While *fnr* was expressed in *A. aromaticum* EbN1^T^ under all tested growth conditions, its genetic localization is not meaningful with respect to the possible members of the FNR regulon. In addition to *fnr*, the genome of *A. aromaticum* EbN1^T^ harbors further genes for regulators belonging to the Fnr/Crp family, including *nnr* and *dnrD* (with only the latter being expressed), whose products are known from pseudomonads to control the expression of *nirS* and *norC*, respectively (18, 48, 49). Accordingly, recognition motifs for FNR-type regulatory proteins were found in the promoter upstream regions of *narK*, *narH*, *nirS*, *norBC*, *nosZ*, and *nosR* (Fig. S3I).

The *nosR* gene is located directly downstream of *nosZ*, and it was found to be expressed under all of the growth conditions in the present study. NosR is a membrane-bound protein that was previously suggested to be involved in the transcriptional control of *nosZ* in P. stutzeri (50). More recently, NosR was implicated as a constituent of a transmembrane protein scaffold-binding NosZ, facilitating the assembly of a nitrate respirasome supercomplex that performs the entire denitrification reaction cascade in P. aeruginosa (51).

The expression of denitrification genes requires activation by the nitrate-responsive, two-component systems NarXL and NarQP (52, 53). *A. aromaticum* EbN1^T^ possesses two *narXL* paralogues, but only the one (*EbN1_C36310/20*) located between *narGHI* and *nosZ* was found to be expressed. It is possible that further regulators, transcription factors such as σ^54^ and small RNAs (54), participate in the transcriptional control of the denitrification genes. The genetic neighborhoods of the assigned sensory/regulatory proteins and their regulatory circuits are illustrated in Fig. S3I.

### Genome revisited and multi-omics data set.

The complete, Sanger-sequenced and manually annotated 4.76 Mbp genome of *A. aromaticum* EbN1^T^ was originally published in 2005 (18). To provide an up-to-date database for the construction of a genome-scale metabolic model, the genome was resequenced and manually reannotated. Resequencing by Illumina (300-fold coverage) revealed just five variants on the chromosome, namely, four SNPs (1194789A→G, 1620654G→A, 4130840A→C, 4195804T→G) and one deletion event (4116523CG→C). In the process of reannotation, current advances specific to the physiology/biochemistry of *A. aromaticum* EbN1^T^ and in the general databases were integrated. Moreover, original, NCBI-based, and current ORF predictions, as well as functional annotations, were reconciled. The revised genome contains 4,642 protein-encoding genes, of which 1,495 represent enzymes with full (1,134) or partial EC numbers. In total, 552 of the predicted enzymes were assigned a new activity, often a more specific one. Also, different activities were predicted based on current enzyme knowledge. Notably, this includes 81 proteins previously classified as “hypothetical proteins”.

*A. aromaticum* EbN1^T^ devotes a rather large share of encoded proteins to enzymatic functions. This is consistent with the experimental results that show that the organism can use carbon sources that have chemical structures that require complex and biochemically demanding degradation pathways in anoxic as well as oxic environments (16). This metabolic versatility is reflected in the number of enzymes that are predicted to modify the redox states of these substances (i.e., 178 dehydrogenases, 47 (de)hydratases, 156 reductases, and 57 oxidases).

Cumulatively, the differential multi-omics data set that was generated across each of the 10 studied growth conditions comprises 4,553 distinct transcripts (covering 95% of the total gene landscape), 2,337 proteins (50% of the encoded proteins), and 139 metabolites (including 70 CoA-esters).

### Genome-scale metabolic model.

**FIG 5:**
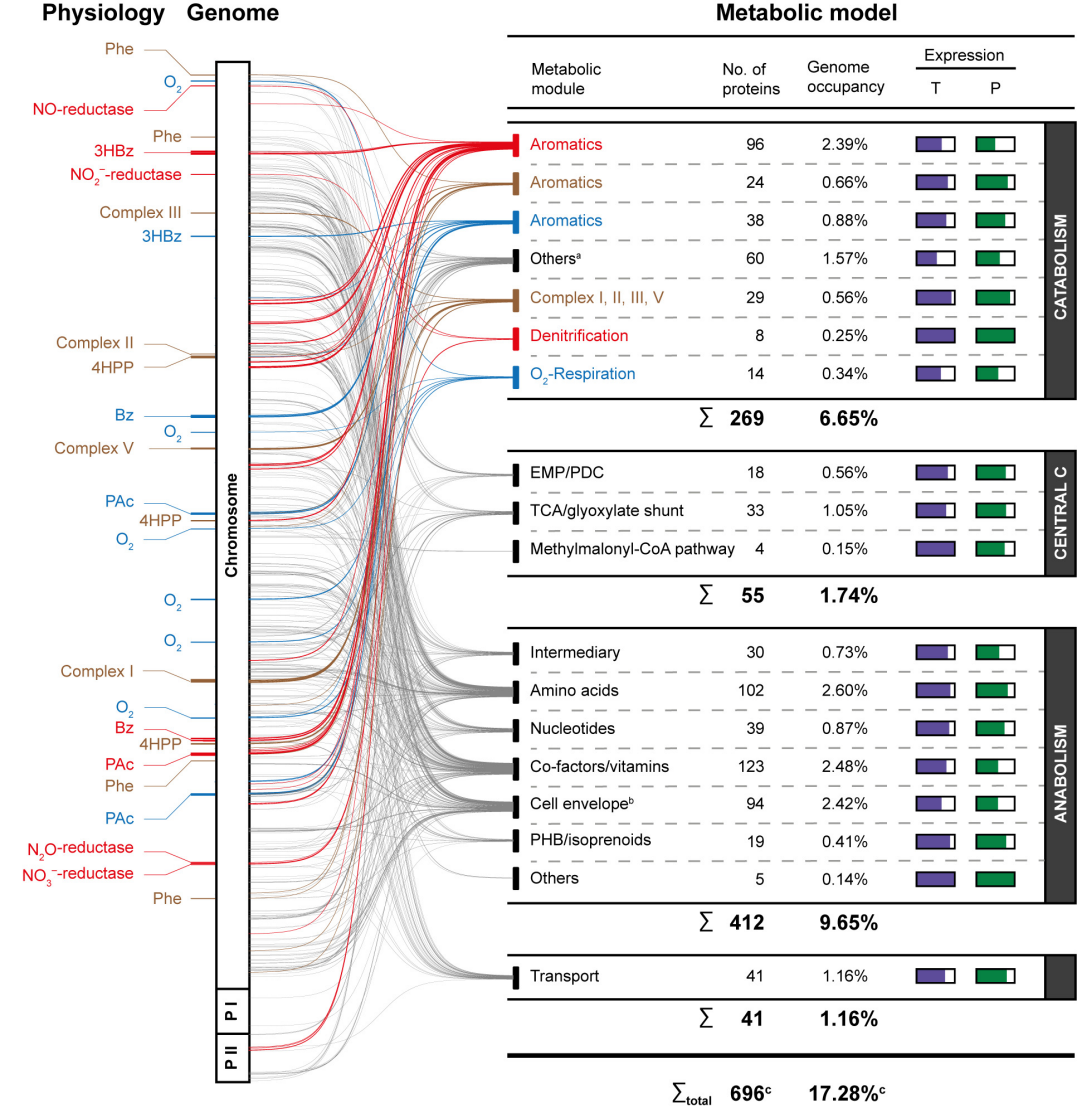
From the reannotated genome to a metabolic model of *A. aromaticum* EbN1^T^. Regarding physiology, the genomic loci of the protein components of the degradation and respiration networks are highlighted. Regarding the genome, the genomic loci of all proteins required for constructing the metabolic model are indicated, with the line thickness reflecting the number of colocalizing genes. For metabolic module-specific illustrations, refer to Fig. S5. Regarding the metabolic model, the figure shows the compilation of the proportional contributions of individual metabolic modules to the overall model and illustrates the degree of gene expression/protein formation within each module, based on the current transcriptomic (T) and proteomic (P) data (Table S1). Ancillary information: ^a^ short-chained organic acids, alcohols and ketones, amino acids; ^b^ phospholipids, peptidoglycan, and LPS; ^c^ nonredundant proteins (i.e., proteins used in more than one metabolic module) are counted only once. The color coding is as in Fig. 2 and Fig 4.

**FIG 6:**
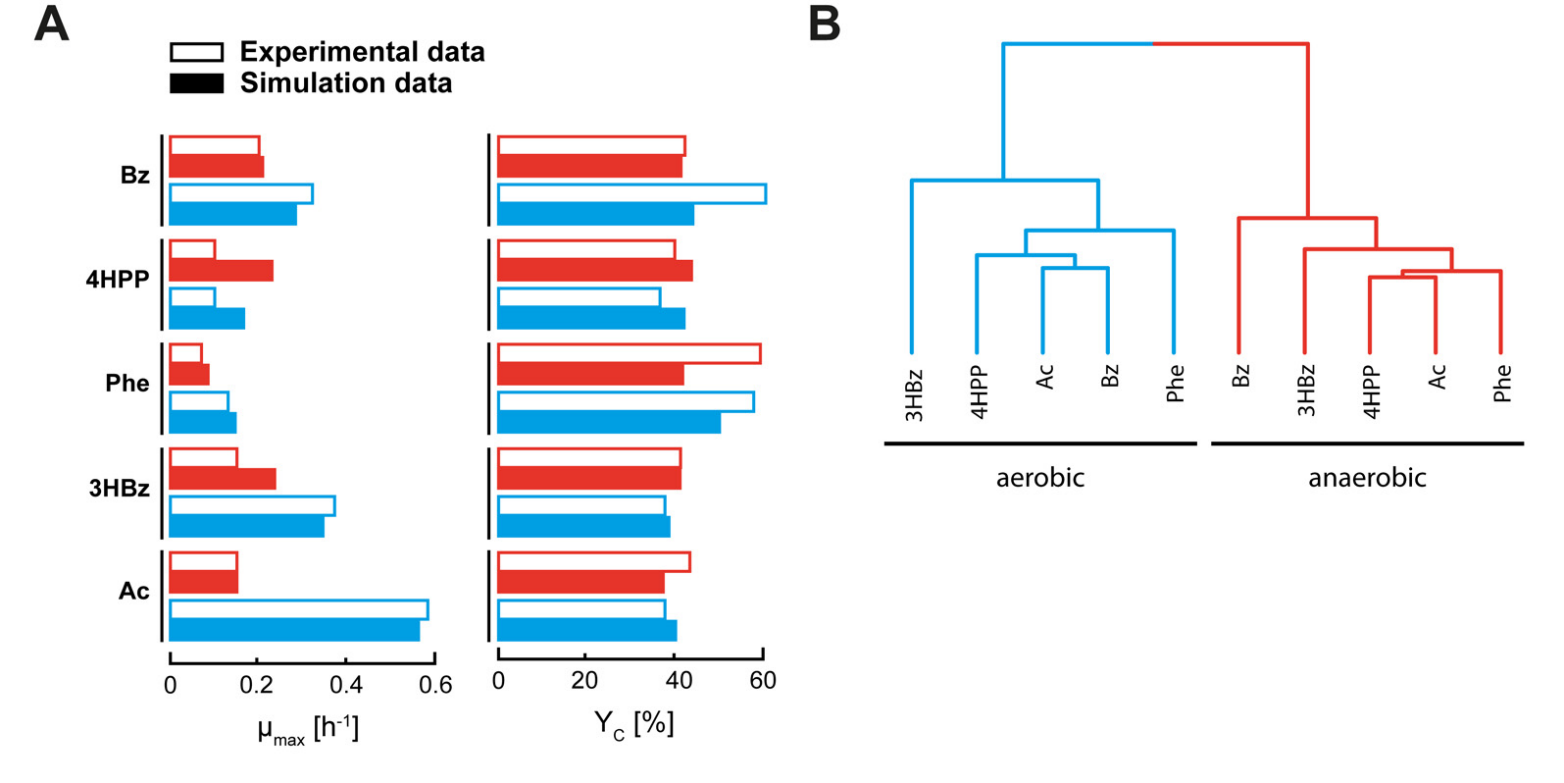
General performance of the metabolic model of *A. aromaticum* EbN1^T^. (A) Simulated data of the 10 growth conditions are compared to the respective experimental data from quantitative growth experiments. (B) Hierarchical clustering of the simulated fluxes for the 10 growth conditions. Further details are provided in Table S3. The abbreviations of the adaptation substrates are as described in the legend of Fig. 2.

**(i) Design and general performance of the metabolic model.** The functional correlations between the aforementioned degradation and respiration networks (Fig. 2 and 4), the reannotated genome, and the metabolic model constructed are displayed in Fig. 5 (for the deconvolution, see Fig. S5). All of the genes involved, including their respective transcript and protein formation, are compiled in Table S1. With the inclusion of boundary, transfer, and definition “reactions”, the model consists of 736 reactions, 655 of which are enzyme-catalyzed functions, and 731 distinct metabolites. Excluding transfer reactions and biomass-forming reactions, the model contains 655 metabolic reactions. In total, the metabolic model comprises 269 and 412 enzymes in the anabolic and catabolic pathways, respectively, together accounting for ~15% of the predicted proteome. Notably, the respective coding genes were mostly found to be expressed, with their protein products formed (Fig. 5). Metabolic fluxes were calculated for aerobic and anaerobic cultivations with acetate and with the aromatic compounds used. While PHB formation is implemented in the metabolic model, experimentally, it could only be measured qualitatively (Fig. S1; Table S1). With the exception of 3-(4-hydroxyphenyl)propanoate (with which an intermediate degradation product was exported) (Fig. S1), the model reproduced the general trends of the different cultivations (Fig. 6A; Table S3 for simulated data, and Fig. S1; Table S3 for experimental data). The calculated biomass yield (C/C) varied between 37% (acetate, anaerobic) and 50% (phenylalanine, aerobic) (Table S3).

10.1128/msystems.00685-22.1 FIG S1 Physiological data (cultivation workflow, reproducibility of cell harvesting, PHB formation, growth with 3HPP, stoichiometric data, OD-CDW correlation). Download FIG S1, PDF file, 1.4 MB. Copyright © 2022 Becker et al. 2022 Becker et al. https://creativecommons.org/licenses/by/4.0/ This content is distributed under the terms of the Creative Commons Attribution 4.0 International license.

10.1128/msystems.00685-22.5 FIG S5 Genomic loci of metabolic modules constituting the metabolic model. Download FIG S5, PDF file, 0.5 MB. Copyright © 2022 Becker et al. 2022 Becker et al. https://creativecommons.org/licenses/by/4.0/ This content is distributed under the terms of the Creative Commons Attribution 4.0 International license.

10.1128/msystems.00685-22.8 TABLE S3 Physiological parameters for anaerobic and aerobic growth. Download Table S3, PDF file, 0.1 MB. Copyright © 2022 Becker et al. 2022 Becker et al. https://creativecommons.org/licenses/by/4.0/ This content is distributed under the terms of the Creative Commons Attribution 4.0 International license.

The activity range of the model's 736 reactions across the simulated cultivation conditions is compiled in Table S4. With the aromatic substrates, almost 50% (346) of them are active under all 8 conditions, including the biosynthesis of the cellular compounds, the TCA cycle, the respiratory chain, gluconeogenesis and the pentose phosphate pathway, assimilatory sulfate reduction, and the transport of H^+^, CO_2_, H_2_O, folate, phosphate, ammonia, sulfate, and adenosylcobalamine.

10.1128/msystems.00685-22.9 TABLE S4 Enzymes constituting the metabolic model. Download Table S4, PDF file, 0.03 MB. Copyright © 2022 Becker et al. 2022 Becker et al. https://creativecommons.org/licenses/by/4.0/ This content is distributed under the terms of the Creative Commons Attribution 4.0 International license.

The model illuminates the extraordinary nature of *A. aromaticum* EbN1^T^ and the flexibility of its metabolism. It allows a deep view into the use of the bacterium's internal pathways, showing the wide range of flux usage in biodegradation and in central metabolism. *A. aromaticum* EbN1^T^ has a small number of enzymes using FAD as a cosubstrate. Instead, it uses ferredoxin (13 enzymes) with its wide range of redox potentials (55), particularly in the degradation pathways of aromatic compounds. Moreover, *A. aromaticum* EbN1^T^ possesses several enzymes that transfer electrons between NAD, NADP, ferredoxin, flavodoxin, ETFs, and ubiquinol, which enable a wide flexibility of metabolic pathways. Whereas under some cultivation conditions, electrons are transferred from ferredoxin to NAD^+^ or NADP^+^, in other cases, the opposite direction is used.

**FIG 7:**
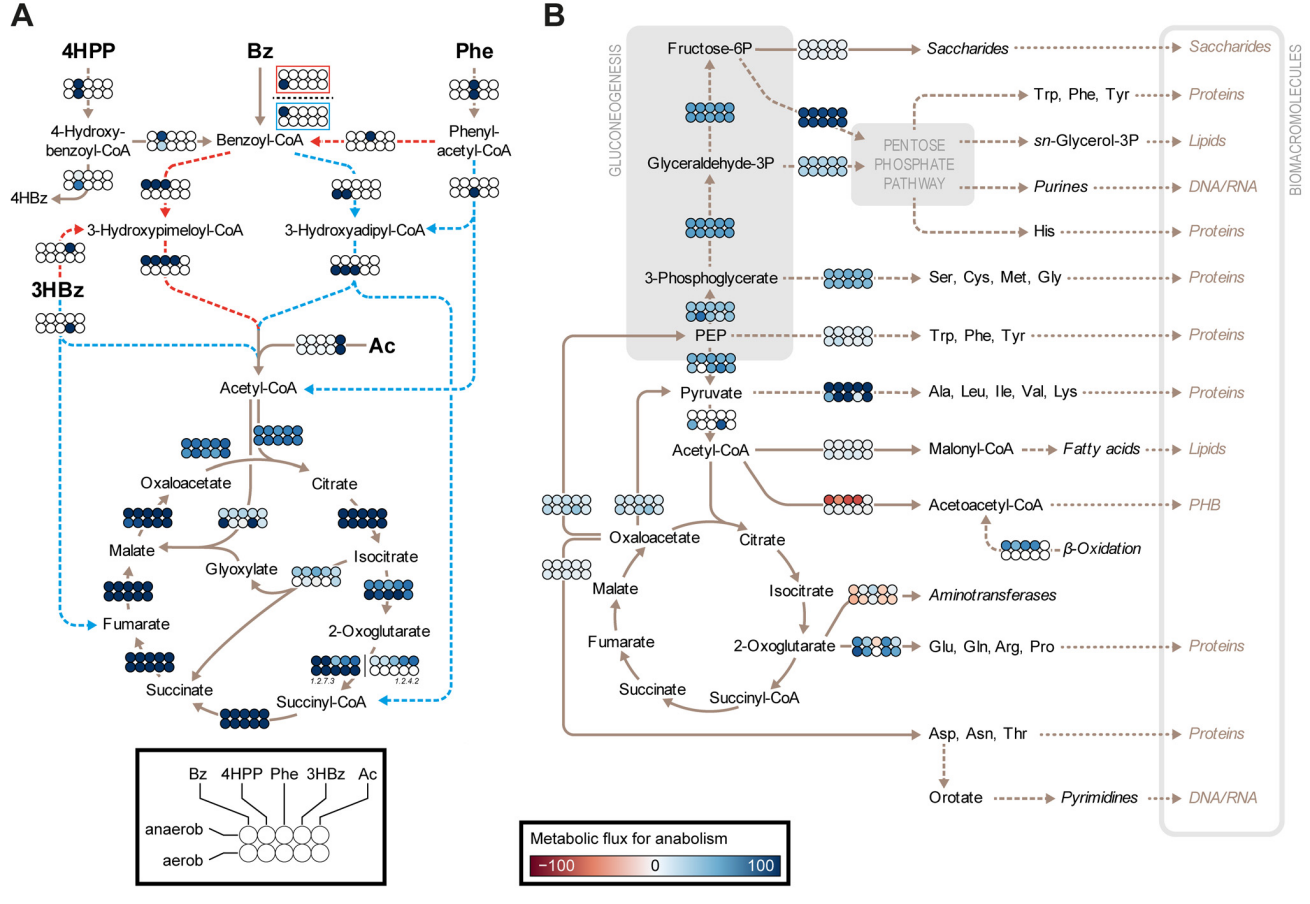
Simulated fluxes. (A) Fluxes through the degradation network studied. The abbreviations of the adaptation substrates are as described in the legend of Fig. 2. (B) Fluxes through the anabolism en route to biomacromolecules. Further details are provided in Table S5.

**(ii) Central metabolism.** The simulated fluxes through the degradation network are illustrated in Fig. 7A (Table S5). While acetate, as the carbon source, enters the TCA cycle after a one-step activation to acetyl-CoA, the different peripheral aromatic degradation pathways end either exclusively in acetyl-CoA, in acetate and succinate, or in pyruvate and fumarate. This leads to different relative fluxes through the TCA cycle versus the glyoxylate shunt, with the usage of the latter avoiding the CO_2_-forming reactions of the TCA cycle. The glyoxylate shunt is not used in the simulations of aerobic growth with 3-hydroxybenzoate or benzoate, but up to 50% of the flux is diverted to the shunt in the simulations of the other conditions.

10.1128/msystems.00685-22.10 TABLE S5 Metabolic model fluxes for catabolism. Download Table S5, XLSX file, 0.02 MB. Copyright © 2022 Becker et al. 2022 Becker et al. https://creativecommons.org/licenses/by/4.0/ This content is distributed under the terms of the Creative Commons Attribution 4.0 International license.

The simulated fluxes through anabolism en route to the synthesis of biomacromolecules are illustrated in Fig. 7B (Table S5). A considerable share of 2-oxoglutarate (22 to 58%) is aminated to feed transaminase reactions in the context of amino acid biosynthesis, while transaminase reactions contribute 18 to 46% to the production of 2-oxoglutarate. Most of the oxaloacetate (55 to 81%) is used for the formation of citrate, up to 38% for the anaplerotic production of phosphoenolpyruvate by the GTP-dependent phosphoenolpyruvate carboxykinase, and up to 12% to generate aspartate. A second anaplerotic reaction, catalyzed by malate dehydrogenase, initiates the conversion of malate to pyruvate via oxaloacetate and phosphoenolpyruvate.

The phosphoenolpyruvate generated is consumed for the production of pyruvate (38 to 69%), for the production of 2-phosphoglycerate (24 to 76%) by phosphopyruvate hydratase (for gluconeogenesis), or for the synthesis of chorismate (biosynthesis of aromatic amino acids). Depending on the carbon source, pyruvate is produced by different reactions: under most conditions, from phosphoenolpyruvate (33 to 98%), by alanine dehydrogenase from alanine, directly from fumarylpyruvate in the degradation pathway for 3-hydroxybenzoate (64%), or by malate dehydrogenase. Then, the pyruvate is converted either to acetyl-CoA by pyruvate dehydrogenase (50 to 86%), and/or to alanine, or is used for reactions that supply intermediates for amino acid biosynthesis.

Missing the enzymes of the oxidative part of the pentose phosphate pathway, the nonoxidative reactions and the gluconeogenesis reactions form intermediates for biomass production (e.g., erythrose-4-phosphate is used for chorismate synthesis).

**(iii) Energy metabolism.** The production rates and usages of metabolites involved in energy transfer and storage, specifically ATP, NADH, NADPH, ferredoxin, and ubiquinol, are different, depending on the carbon source. ATP is produced during respiratory energy conservation by H^+^-transporting ATP synthase (76 to 93%), in the TCA cycle from succinyl-CoA by succinate-CoA ligase (5 to 20%), and in the EMP pathway from phosphoenolpyruvate by pyruvate kinase (2 to 8%). ATP is used in 77 different reactions, with growth- and nongrowth-associated maintenance consuming 35 to 62% and 4 to 27%, respectively, the production of GTP demanding 2 to 12%, and growth with acetate requiring 34 to 37% to fuel acetate-CoA ligation as the first degradation step (note that 3 alternative enzymes are encoded).

NADH, another energy-storage metabolite, is produced by 22 different reactions and is consumed by 24 reactions. It is mainly produced in the TCA cycle by malate dehydrogenase (33 to 56%), by pyruvate dehydrogenase (5 to 30%), during aerobic growth with 3-hydroxybenzoate, by electron transfer from reduced ferredoxin (6 to 45%), and by a number of dehydrogenase reactions. Most NADH (87 to 96%) is oxidized by the respiratory NADH dehydrogenase, except for the 23% required by the aerobic conversion of 3-hydroxybenzoate to gentisate.

In contrast to typical carbohydrate-consuming organisms, the main sources of NADPH in *A. aromaticum* EbN1^T^ are the TCA cycle reaction catalyzed by isocitrate dehydrogenase, which produces 40 to 99% of the NADPH, as well as reactions in the catabolic pathways of phenylacetate and benzoate. NADPH is produced by 7 reactions and is consumed by 45 reactions (mostly for biosynthetic reactions and for electron transfer to NAD^+^).

**(iv) General features.** The strongest variations between the different simulations are observed for the transfer fluxes of H^+^, nitrate, CO_2_, oxygen, and water, as well as for the respiratory chain and TCA cycle reactions. Together, they are responsible for more than 80% of the total variation between the maximal and minimal flux of the specific reactions. A hierarchical clustering of the fluxes shows a clear distinction between oxic and anoxic conditions, with the substrate condition 3-hydroxybenzoate presenting as an outlier in both clusters (Fig. 6B), which is concordant with the NMF analyses of the degradation network’s gene expression.

## CONCLUSIONS

By combining multi-omics experiments with physiological data and a metabolic model, we have established an advanced study platform for an organism that differs significantly from other well-studied bacteria, such as Escherichia coli or Bacillus subtilis. Our study has significantly increased our understanding of how *A. aromaticum* EbN1^T^ achieves its metabolic flexibility. The model promotes a detailed, quantitative understanding of the interactions between the environmental conditions (oxic/anoxic), carbon source, regulation, transport, and metabolism, as well as energy conservation in *A. aromaticum* EbN1^T^. It became clear that the transcriptome and proteome often do not correlate directly; specifically, proteins are present in higher concentrations in the proteome analysis, despite the low mRNA abundance. It was shown by metabolome analysis that metabolic processes are often mainly linearly led to the central metabolism but sometimes also include parallel pathways or dead ends. For similar substrates, *A. aromaticum* EbN1^T^ uses parallel pathways to the TCA cycle with different entry points. The analysis of CoA-activated intermediates allowed for a clear identification of the degradation step when the consecutive steps of conversion were dependent on CoA activation. Finally, a high-quality, state-of-the-art genome annotation was accomplished. The fact that the metabolic model is able to reproduce both the successful use of simple and challenging substrates and degradation pathways under oxic and anoxic conditions makes it suitable for predictions.

The concurrent formation of the enzymatic equipment for the anaerobic as well as aerobic degradation of toluene was recently observed for betaproteobacterial Georgfuchsia toluolica G5G6^T^, which is capable of using Fe(III) and Mn(IV) in addition to nitrate as alternative electron acceptors (56, 57). Whereas anaerobic toluene degradation by *G. toluolica* G5G6^T^ involves known benzylsuccinate synthase and class I ATP-dependent benzoyl-CoA reductase (as in *A. aromaticum* EbN1^T^), the aerobic route proceeds via the monooxygenase-catalyzed conversion of toluene to *m*-cresol and 3-methylcatechol, followed by dioxygenase-catalyzed ring opening (57). Similarly, most transcripts and proteins constituting the degradation network for the monoaromatic compounds studied under anoxic and oxic conditions were simultaneously formed by *A. aromaticum* EbN1^T^ (and in related *A. aromaticum* pCyN1; [58]). Considering these congruent observations, the notion emerges that facultative anaerobic degradation specialists do not strictly differentiate between anoxic and oxic conditions on a sensory/regulatory level, as known, for example, from standard bacteria, such as E. coli. Rather, they apparently keep the degradation pathways “open”, possibly to instantaneously exploit any combination of organic substrate and electron acceptor as it becomes available in a dynamically changing environment. However, this may come at the price of inactivating the O_2_-sensitive enzymes of the anaerobic degradation routes (e.g., benzoyl-CoA reductase), or this may require special, yet unknown, mechanisms of protection against the oxidative damage of these key enzymes. Nevertheless, the anoxia/oxia transition proved to be a key determinant for physiological adaptation, as revealed by both approaches (i.e., differential multi-omics and the genome-scale metabolic model).

## MATERIALS AND METHODS

### Bacterial strain and general cultivation conditions.

Aromatoleum aromaticum EbN1^T^ was originally isolated with ethylbenzene under nitrate-reducing conditions from freshwater mud (59), maintained in the laboratory, and recently deposited at the Deutsche Sammlung von Mikroorganismen und Zellkulturen (DSMZ), Braunschweig, Germany, under the culture collection number DSM 19018^T^ (16). *A. aromaticum* EbN1^T^ was cultivated under nitrate-reducing conditions in a defined ascorbate-reduced and bicarbonate-buffered mineral medium, as well as under oxic conditions in essentially the same mineral medium (devoid of ascorbate and nitrate) at 28°C, as previously described (59). The following five growth substrates, added from aqueous stock solutions that were sterilized by filtration, were used (in alphabetical order, with the concentrations given in parentheses): acetate (8 mM), benzoate (4 mM anaerobic, 2 mM aerobic), 3-hydroxybenzoate (4 mM anaerobic, 2 mM aerobic), 3-(4-hydroxyphenyl)propanoate (2 mM anaerobic, 4 mM aerobic), and phenylalanine (4 mM anaerobic, 2 mM aerobic). For the anaerobic cultivation, 7 mM nitrate was provided, except for cultures with phenylalanine, which received 10 mM nitrate. Growth was monitored by measuring the optical density (OD) at 660 nm (UVmini-1240 spectrophotometer; Shimadzu, Duisburg, Germany). All of the chemicals were of analytical grade.

### Growth adaptation, cultivation, and harvesting for omics analysis and growth stoichiometry.

*A. aromaticum* EbN1^T^ was adapted to each of the 10 growth conditions (five substrates, each anaerobic and aerobic) over at least five passages (80 mL culture volume). Cultures were inoculated with 2 or 5% (vol/vol) of an actively growing (½ OD_max_), adapted preculture. To provide sufficient cell material for omics profiling and for the determination of growth stoichiometry, cultivation was performed in 500 mL flat-bottomed glass bottles (400 mL culture volume) that were sealed with butyl rubber stoppers under an N_2_/CO_2_-atmosphere (anoxic condition) or in 1 L round glass bottles (400 mL culture volume) sealed with butyl rubber stoppers under an atmosphere of sterile air (oxic condition). In total, 25 parallel 400 mL cultures were run per growth condition: 3 for transcriptomics, 3 for soluble/membrane proteomics, 8 for metabolomics (4 for GC and 4 for the high-performance liquid chromatography (HPLC)-based analyses), and 11 each for the determination of growth stoichiometry (5, including 2 negative controls for the growth curve and 3 for the cellular dry weight and the elemental composition of the biomass at an OD of 0.3 and at OD_max_). All of the samples for the omics analyses were harvested at an OD of 0.3. The harvesting for the three different omics approaches is described in the following. In the cases of anaerobic cultures, the centrifuge beakers were flushed with N_2_ to reduce the impact of O_2_ during harvesting.

**(i) Harvesting for transcriptomics.** The entire 400 mL cultures were centrifuged (14,334 × *g* for 20 min at 4°C). The resultant cell pellet was resuspended in 1.5 mL of RNAprotect (Qiagen, Hilden, Germany), transferred into a 2 mL microcentrifuge tube, incubated for 10 min at ambient temperature, and centrifuged again (20,000 × *g* for 20 min at 4°C). The final cell pellets were shock frozen in liquid N_2_ and stored at −80°C for further analysis.

**(ii) Harvesting for proteomics.** Cultures were harvested for proteomic analyses as previously described (60). Essentially, the complete 400 mL cultures were centrifuged (14,334 × *g* for 30 min at 4°C). The pellets were washed in 250 mL of washing buffer (100 mM Tris/HCl, 5 mM MgCl_2_ × 6 H_2_O, pH 7.5) and re-suspended in 0.8 mL of the same washing buffer. Following a second centrifugation (20,000 × *g* for 10 min at 4°C), the cell pellets were shock frozen in liquid N_2_ and stored at −80°C for further analysis.

**(iii) Harvesting for metabolomics.** The entire 400 mL cultures were centrifuged (17,700 × *g* for 15 min at 4°C), and the subsequent steps were adjusted to the designated analytical approach. For the HPLC-MS analyses, the cell pellets were immediately resuspended in 2 mL of methanol containing ~0.7 nM biochanin A and transferred in portions of 1 mL into Precellys tubes (Bertin Corp., Rockville, MD, USA) containing 600 mg glass beads, sized 70 to 119 μm (Kuhmichel Abrasive GmbH, Ratingen, Germany). The suspensions were then shock frozen in liquid N_2_ and stored at −80°C for further analysis. For the GC-MS analyses, the cell pellets were immediately washed with 100 mL NaCl (0.9% [wt/vol], anoxic for anaerobic cultures) and centrifuged (17,700 × *g* for 15 min at 4°C). The resultant pellets were immediately resuspended in 2 mL of MeOH containing 0.02 to 0.05 mM ribitol, transferred in portions of 1 mL into Precellys tubes (see above), shock frozen in liquid N_2_, and stored at −80°C for further analysis.

### Growth stoichiometry.

To record the growth curves for each tested growth condition, duplicate 2 mL samples were retrieved in short intervals from each replicate culture using sterile syringes (flushed with N_2_ in cases of anaerobic cultivation) and transferred to microreaction tubes. After measuring the OD, the samples were centrifuged (20,000 × *g* for 10 min at 4°C), and the resultant supernatants were stored at −20°C for the further analysis of the substrate depletion by means of HPLC and IC. Prior to these analyses, the thawed samples were filtered (pore size, 0.2 μm; regenerated cellulose[RC]; Chroma Globe, Kreuzau, Germany).

**(i) Quantification of aromatic compounds.** The depletion of aromatic growth substrates was measured using an HPLC instrument (UltiMate 3000; Thermo Fisher Scientific, Germering, Bavaria, Germany) equipped with a Thermo Hypersil Gold column (C_18_, 150 × 1 mm, 2.6 μm bead size; Thermo Fisher Scientific) and a rapid-separation (RS) diode array detector (Thermo Fisher Scientific), as described previously (61). The system was operated at 40°C with a flow rate of 100 μL/min. The 20 min gradient of eluent A (5% [vol/vol] acetonitrile in H_2_O with 0.01% [vol/vol] H_3_PO_4_ [85%]) and eluent B (90% [vol/vol] acetonitrile in H_2_O with 0.01% [vol/vol] H_3_PO_4_ [85%]) was held constant for 2.5 min at 3% B and was followed by a 4 min linear ramping to 65% B and a constant level of 3% B for 9 min. Benzoate was detected at 229 nm with a retention time of 9.32 min and a dynamic range from 5 nM to 50 μM. 3-hydroxybenzoate was detected at 236 nm with a retention time of 8.3 min and a dynamic range from 1 μM to 1 mM. 3-(4-hydroxyphenyl)propanoate was detected at 236 nm with a retention time of 9.1 min and a dynamic range from 0.1 μM to 2.5 mM. Phenylalanine was detected at 210 nm with a retention time of 8.3 min and a dynamic range from 10 μM to 1 mM.

**(ii) Quantification of acetate.** The depletion of acetate was measured by HPLC (UltiMate3000) using a Eurokat H separation column (8 × 300 mm, pore size 10 μm; Knauer, Berlin, Germany) at an oven temperature of 75°C, an isocratic 5 mM H_2_SO_4_ eluent, a flow rate of 1.2 mL/min, and an injection volume of 50* *μL. Acetic acid was detected at 210 nm with a retention time of 8.0 min and a dynamic range from 25 μM to 1 mM.

**(iii) Quantification of nitrate and nitrite.** Both anions were analyzed via isocratic ion chromatography (IC) (ICS-1100; Thermo Fisher Scientific) using a carbonate eluent (9 mM Na_2_CO_3_) and an analytical IonPac AS9-HC column (4 × 250 mm; Thermo Fisher Scientific). This analytical column was protected from particles by two anion neutral guard columns (IonPac AG9-HC [4 × 50 mm], Guard & IonPac NG1 [4 × 35 mm]; Thermo Fisher Scientific). The samples were diluted 1:5 with distilled water in a volume of 500 μL, transferred into 0.5 mL polyvials with filter caps, and injected by an AS-DV autosampler (Thermo Fisher Scientific). The separation of nitrate and nitrite occurred at a flow rate of 1 mL/min and resulted in retention times of 13.3 min and 8.9 min, respectively. For anaerobic cultivations with acetate, detection was at 210 nm with a dynamic range from 10 μM to 5 mM. For all other growth conditions, a conductivity detector was used (DS6 Heated Conductivity Cell; Thermo Fisher Scientific), yielding a dynamic range from 50 μM to 5 mM. An electrolytic suppressor (4 mm) operated at 45 mA (ASRS 300; Thermo Fisher Scientific) was connected upstream of the conductivity detector to trap the eluent ions, thereby decreasing the background conductivity.

**(iv) Determination of cellular dry weight and elemental composition.** Separate triplicate 400 mL cultures were conducted for each growth condition to measure the cellular dry weight and elemental composition at an OD of 0.3 and at OD_max_. In each case, 100 mL of culture broth was retrieved and subsampled (4 × 2 mL) for the OD, total cell numbers (Thoma counting chamber; Glaswarenfabrik Karl Hecht GmbH & Co. KG, Sondheim vor der Rhön, Germany), and HPLC/IC measurements to confirm congruent growth stages and substrate consumption, compared to the detailed growth curves. Following the centrifugation of 3 × 10 mL culture broth (14,334 × *g* for 20 min at 4°C), the pellets were each resuspended in 5 mL of 0.9% (wt/vol) NaCl. The centrifugation beakers were washed with 5 mL of a 0.9% NaCl solution. The combined ~10 mL cell suspensions were transferred to new beakers and centrifuged again (14,500 × *g* for 20 min at 4°C). The resultant cell pellets were each resuspended in 1 mL of 0.9% NaCl and were then transferred in 400* *μL aliquots into three predried, preweighed 1.5 mL tubes. The cellular dry weight was then determined via gravimetric analysis of the tubes after drying to constant weight at 60°C (the dry weight of tubes with only 1 mL of 0.9% NaCl was subtracted). The dried cells were then used to determine the elemental composition (C, H, N, and S) using a vario EL cube (Elementar Analysensysteme, Hanau, Germany). This method involves high-temperature oxidation (up to 1,800°C), chromatographic separation, and detection by heat conductivity (62). The O content (expressed as weight percent [wt%]) of the dried cells was calculated by subtracting the experimentally determined C, H, and N contents (in weight percent) as well as the estimated ash content (including S) of 12.03 wt% from the 100 wt% (63).

**(v) Calculations.** Growth physiological parameters, such as the growth rate (μ), doubling time (t_D_), biomass-specific carbon consumption rate (*q_C_*_/_*_X_*), and biomass-specific reducing equivalent consumption rate (*q*_[_*_H_*_]/_*_X_*) were essentially calculated as described previously (64, 65). For the correlation of the OD with the cellular dry weight and the total cell numbers, refer to Fig. S1. The stoichiometric equations of the substrate metabolism to estimate the dissimilatory and assimilatory shares are compiled in Table S3.

### Resequencing of genome.

The genome of *A. aromaticum* EbN1^T^ was re-sequenced using whole-genome sequencing, short-read mapping, and variant calling. The libraries for the whole-genome sequencing on the Illumina platform were prepared from extracted genomic DNA, applying the Nextera XT DNA Library Preparation Kit (Illumina, San Diego, USA) with modifications (66). The samples were subsequently sequenced on an Illumina NextSeq 500. The quality control of the Illumina reads was performed using fastp (67). The genome re-sequencing was performed via the mapping of Illumina short reads onto the complete genome data (GenBank accession numbers CR555306.1, CR555307.1, and CR555308.1) using BWA (68), variant calling by VarScan 2.3.6 (69), and the final establishment of a new consensus sequence by AlternateReferenceMaker (https://github.com/JHartlich/AlternateReferenceMaker).

### Reannotation of genome.

To improve the functional annotation of the genome of *A. aromaticum* EbN1^T^, the original ORF prediction and annotation (18) were completely revised using the following approaches: (i) specific functional refinements based on our previous proteomic and genetic studies, as well as the current state of the relevant literature; (ii) updating the predictions on enzyme functions by screening against the BRENDA EnzymeDetector (70), which delivers rated enzyme function predictions based on a combination of manual assignments in the BRENDA experimental database, predictions from Uniprot, KEGG, PATRIC, and BLAST hits, and a sequence pattern analysis with a weighting scheme that is based on the calculated correctness of the different predictors used; (iii) updating the predictions of putative transporters benefitted from a relevant study by Tamang et al. (71), which is complemented by the current screening against the most recent version of the transporter classification database (TCDB) (72); (iv) updating the predictions of putative sensory/regulatory proteins by consulting the InterPro database (73); and (v) revisiting proteins of unknown function by conducting a new BLAST analysis (74), as implemented on the ExPASy-server (75).

### Transcriptomics.

**(i) RNA sequencing and expression-level calculation.** Transcriptomic analyses were conducted as commissioned work by the team of Robert Geffers (HZI, Braunschweig, Germany). The quality and integrity of the total RNA were controlled with a 2100 Bioanalyzer (Agilent Technologies, Waldbronn, Germany). The RNA sequencing library was generated from 1,000 ng of total RNA after rRNA depletion using RiboZero Plus (Illumina, San Diego, CA, USA), followed by a ScriptSeq v2 RNA-Seq Library Preparation Kit (Epicentre, an Illumina company), according to the manufacturers’ protocols. The libraries were sequenced on an Illumina HiSeq2500, using a TruSeq SBS Kit v3-HS (50 cycles, single ended run) with an average of 3 × 10^7^ reads per sample (76). The expression levels were calculated from the mean values of three biological replicates after normalization, alignment, and assembly, using the open-source software Rockhopper (77, 78). For the raw data counts, an estimated cutoff-value of 50 was set. This led to the inclusion of all expression values larger than 1 into the scaling and interpretation of the transcriptomic data sets.

**(ii) Transcriptional variance analyses.** Transcriptomic data were subjected to nonnegative matrix factorization (NMF) to identify metagene expression types (components) (40–42). NMF modeling was performed with the function sklearn.decomposition.NMF from the Python machine-learning package “scikit-learn” (version 0.24.1, Python 3.7). In short, NMF decomposes the original transcript matrix **T** into a product of two simpler matrices **W** and **S** such that $\tilde{T}=W\cdot S$ approximates the original matrix **T**, which, in index notation, can be written as:

$$\overset{m}{\underset{k=1}{\sum }}W_{\mathrm{ik}}S_{\mathrm{kj}}={\tilde{T}}_{\mathrm{ij}}\approx T_{\mathrm{ij}},$$

where $W_{\mathrm{ik}}$ is the weight of the *i*^th^ gene in the *k*^th^ metagene expression type, $S_{\mathrm{kj}}$ is the metagene expression score (contribution) of the *j*^th^ growth condition to the *k*^th^ metagene, and $T_{\mathrm{ij}}\;$ is the expression level of the *i*^th^ gene in the *j*^th^ growth condition. All the expression levels $T_{\mathrm{ij}}$ in any given growth condition *j* are normalized by the total expression level summed over all *N* of the genes in that condition, (i.e., $\sum_{i=1}^{N}T_{\mathrm{ij}}=1$). The distribution of the copy numbers of all 4,100 of the transcripts in any growth condition was found to follow a lognormal distribution such that the total variance of the transcriptome was dominated by the upper tail of the copy number distribution (i.e., by the highly expressed genes). Therefore, we log-transformed the original copy number $c_{\mathrm{ij}}$ in the global data set, that is, $T_{\mathrm{ij}}=\text{log}(1+c_{\mathrm{ij}})$, to account for the majority of genes (which, by definition, have intermediate expression levels). To find the adequate number *m* of metagene expression types, we require that the approximative matrix $\tilde{T}$ shall explain at least 90% of the condition-wise variance (along a given growth/substrate condition) for each of the *M *= 10 growth conditions, which resulted in *m *= 5 components explaining 95% and 99% of the total variance of the transcript matrix for the degradation network and the global data set, respectively. The transposed scores matrix is presented as a heat map in Fig. 3. The metagene expression types are shown in Fig. S4.

### Proteomics.

For the 10 growth conditions, the total soluble and membrane protein-enriched fractions were prepared and analyzed for three biological replicates each.

**(i) Soluble protein fraction.** The cells were resuspended in 1 mL of lysis buffer (7 M urea, 2 M thiourea, 30 mM Tris, 4% [wt/vol] CHAPS, pH 8.5) and disrupted using the PlusOne Sample Grinding Kit (GE Healthcare, Munich, Germany) as previously described (79). The suspension was centrifuged twice (100,000 × *g* for 1 h at 4°C) prior to protein quantification, according to the method described by Bradford (80). Whole-cell shotgun analysis was carried out as previously described (62). Tryptic peptides were separated by nanoLC (UltiMate3000 nanoRSLC; Thermo Fisher Scientific) operated in a trap-column mode (C_18_, 2 cm, 3 μm bead size, 75 μm inner diameter; Thermo Fisher Scientific), and equipped with a 25 cm separation column (C_18_, 2 μm bead size, 75 μm inner diameter; Thermo Fisher Scientific), applying a 280 min linear acetonitrile gradient. The eluent was continuously analyzed by an online-coupled ion-trap mass spectrometer (amaZon ETD; Bruker Daltonik GmbH, Bremen, Germany) with the acquisition of 10 MS/MS spectra of the most intense doubly (or more) charged ions per full-scan MS (mass range of 400 to 1,400 *m/z*), applying subsequent precursor exclusion for 0.2 min.

**(ii) Membrane-protein enriched fraction.** The cell pellets were resuspended and disrupted using a French press (Sim-Aminco Ltd., Rochester, NY, USA) as previously described (62). The cell extracts were treated with ice-cold carbonate, and the proteins were solubilized using hot SDS. The protein content was determined with the RC-DC assay (Bio-Rad), and protein separation was achieved using 12.5% SDS mini gels (10 × 7 cm; Bio-Rad). Following Coomassie staining (62), each sample lane (10 μg protein load) was divided into 8 gel slices, and each slice was cut into smaller pieces (about 1 mm^3^) prior to washing, reduction, alkylation, and tryptic digestion (62). The analysis of tryptic peptides via nanoLC-ESI-MS/MS was as described above for the soluble protein fraction, except for the application of a 120 min linear acetonitrile gradient.

**(iii) Protein identification.** Proteins were identified via the ProteinScape platform (version 3.1; Bruker Daltonik GmbH), using an in-house Mascot server (version 2.3; Matrix Science Ltd., London, UK) that was based on the translated genome of *A. aromaticum* EbN1^T^ (18). A target decoy strategy was applied, using the following settings: taxonomy, all entries; enzyme, trypsin; may missed cleavage, 1; fixed modification, carbamidomethyl (C); variable modification, oxidation (M); peptide mass tolerance, 0.3 Da; fragment mass tolerance, 0.4 Da; mass values, monoisotopic; significance threshold, 0.05; instrument type, ESI-TRAP; and inclusion of peptide decoy with a false discovery rate of 1%. The search results of the analyses of the three biological replicates were compiled.

### Metabolomics.

**(i) Extraction of small polar metabolites.** The samples were thawed on ice before homogenization at −10°C for 3 cycles at a speed of 6,800 rpm for 30 s with breaks of 30 s, using a Precellys 24 homogenizer (Bertin Instruments, Montigny-le-Bretonneux, France). Afterwards, 400 μL of water were added to the sample, and this was followed by a mixing step of 2 min at 2,000 rpm (MixMate; Eppendorf, Hamburg, Germany). Then, 250 μL of chloroform were added, and the sample was mixed again for 2 min at 2,000 rpm prior to centrifugation (10,000 rpm for 10 min at 4°C). Subsequently, the polar phase was transferred to 2 mL reaction tubes and dried at 14°C under a vacuum. The samples were stored at −20°C until derivatization for the GC-EI-MS analysis. For each growth condition, 4 biological samples with 2 technical replicates each were analyzed.

**(ii) Derivatization for GC-MS measurement.** For every GC-MS analysis, a chemical derivatization was employed. In the first step, 40 μL pyridine with 20 mg/mL methoxyamine hydrochloride were added and incubated for 90 min at 30°C under constant agitation (1,000 rpm). Second, 60 μL of MSTFA were added, mixed, and incubated for 30 min at 37°C, followed by 120 min at 25°C under constant agitation.

**(iii) GC-EI-MS analysis of metabolites.** Samples of the oxic condition were analyzed using the Accu TOF JMS-T100GC (JEOL GmbH, Freising, Germany), coupled to a MPS 2 XL autosampler (Gerstel GmbH & Co KG, Mühlheim an der Ruhr, Germany) and an 6890N Agilent GC (Agilent Technologies, Santa Clara, CA, USA) in splitless mode. The exometabolome samples and those of the anoxic condition were analyzed using a DSQ II MS (Thermo Fisher Scientific), coupled to an autosampler 3000 (Thermo Fisher Scientific) and a Thermo GC Ultra (Thermo Fisher Scientific). The data were processed with a metabolite detector (81) and were normalized to the dry mass of the cells and the internal standard, ribitol. The data on the small polar metabolites are compiled in Table S2.

**(iv) Extraction and LC-MS/MS analysis of CoA-thioesters.** The samples were thawed on ice before mechanical cell lysis (Precellys 24 homogenizer), and the concentration and purification of CoA-thioesters via solid phase extraction as well as the following LC-MS/MS analysis were performed as previously described (82), using the same LC-MS device. For each growth condition, 4 biological samples with 2 technical replicates each were analyzed.

**(v) Reliability of CoA-thioester identification.** Since commercially available CoA-thioesters are rare, many of the CoA-thioesters that were relevant for this study were identified on the basis of their absolute masses, their isotope patterns, their retention times, and the existence of fragments of the CoA residue. In some cases, the assignment of CoA-thioesters could be enhanced via comparison with the CoA measurements of other organisms that share metabolic routes with *A. aromaticum* EbN1^T^. Information concerning the reliability of each identified CoA-thioester is given in Table S2.

### Reconstruction and analysis of the metabolic model *i*AA835.

The stoichiometric model was reconstructed bottom-up, based on the genome reannotation presented in this study and on the application of the integrative reaction lookup service of the Metano Modeling Toolbox (MMTB) (83). The reaction names were manually adjusted to include information on pathways and enzymes, as well as reaction identifiers from public databases. We included locus tags that encode the corresponding enzymes in the comments for each reaction. Transcriptome and proteome data were integrated to revise the model. The macromolecular biomass composition was taken from the model proteobacterium Escherichia coli (84). The fatty acid composition was taken from previous reports (16, 85). The composition of the DNA and RNA was estimated using the genome sequence of *A. aromaticum* EbN1^T^ (18), as described in (86). Accordingly, the relative protein composition was calculated using all predicted protein sequences. The pool of soluble compounds was made up from all of the compounds that are essential for growth and would not otherwise be produced by the model, including vitamins, cofactors, and reduction equivalents. The maintenance energy was taken from the model proteobacterium Escherichia coli K-12 (87).

The model was analyzed using MMTB. A flux balance analysis (FBA) was performed using the maximum biomass yield as the objective function. Metabolite-centric fluxes through metabolic nodes were analyzed via split-ratio analysis.

### Data availability.

The revised genome annotation of *A. aromaticum* EbN1^T^, as well as the transcriptomic, proteomic, and metabolomic data, have been deposited at FAIRDOMHub (https://doi.org/10.15490/FAIRDOMHUB.1.INVESTIGATION.534.1). The metabolic model is deposited at MMTB under the name *i*AA835 (mmtb.brenda-enzymes.org/models/view/15).
