# Supplementary material for: Systems Biology of Aromatic Compound Catabolism in Facultative Anaerobic Aromatoleum aromaticum EbN1T
Source: mSystems. 2022 Nov 29;7(6):e00685-22. doi: 10.1128/msystems.00685-22 (PMC9765128; doi:10.1128/msystems.00685-22)
Supplement: FIG S2 [file msystems.00685-22-s0002.pdf]

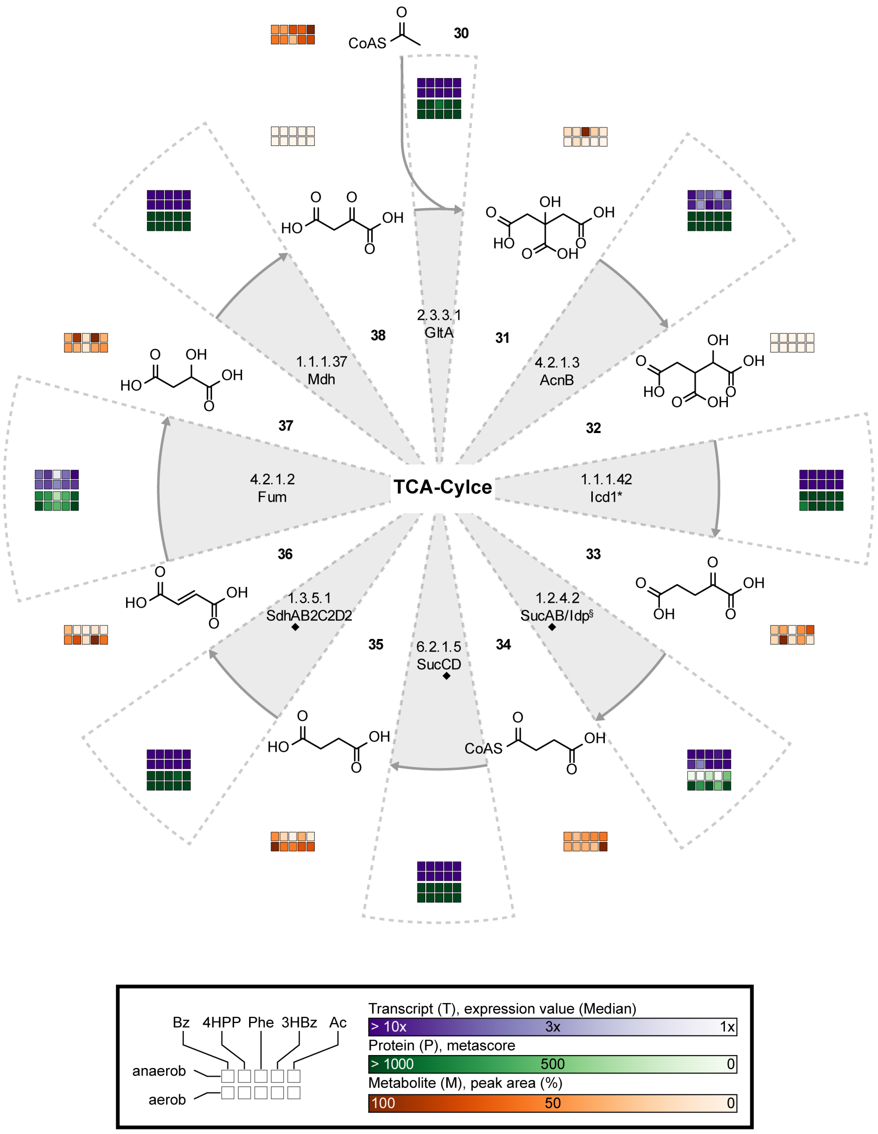

FIG S2 TCA-cycle superimposed with differential multi-OMICS data. ♦, selected enzyme subunit for which transcript and protein abundances are shown. *, paralogous Icd (EbN1_C04460) also displayed high levels of transcript and protein abundances. §, a KorCAB paralog (EbN1_C26200/10/20), transcribed and formed at high level, is assumed to provide reduced ferredoxin for benzoyl-CoA reductase. Underlying transcriptomic, proteomic and metabolomic data, as well as the compound names are provided in Table S1 and S2.
