# Supplementary material for: Systems Biology of Aromatic Compound Catabolism in Facultative Anaerobic Aromatoleum aromaticum EbN1T
Source: mSystems. 2022 Nov 29;7(6):e00685-22. doi: 10.1128/msystems.00685-22 (PMC9765128; doi:10.1128/msystems.00685-22)
Supplement: FIG S3 [file msystems.00685-22-s0003.pdf]

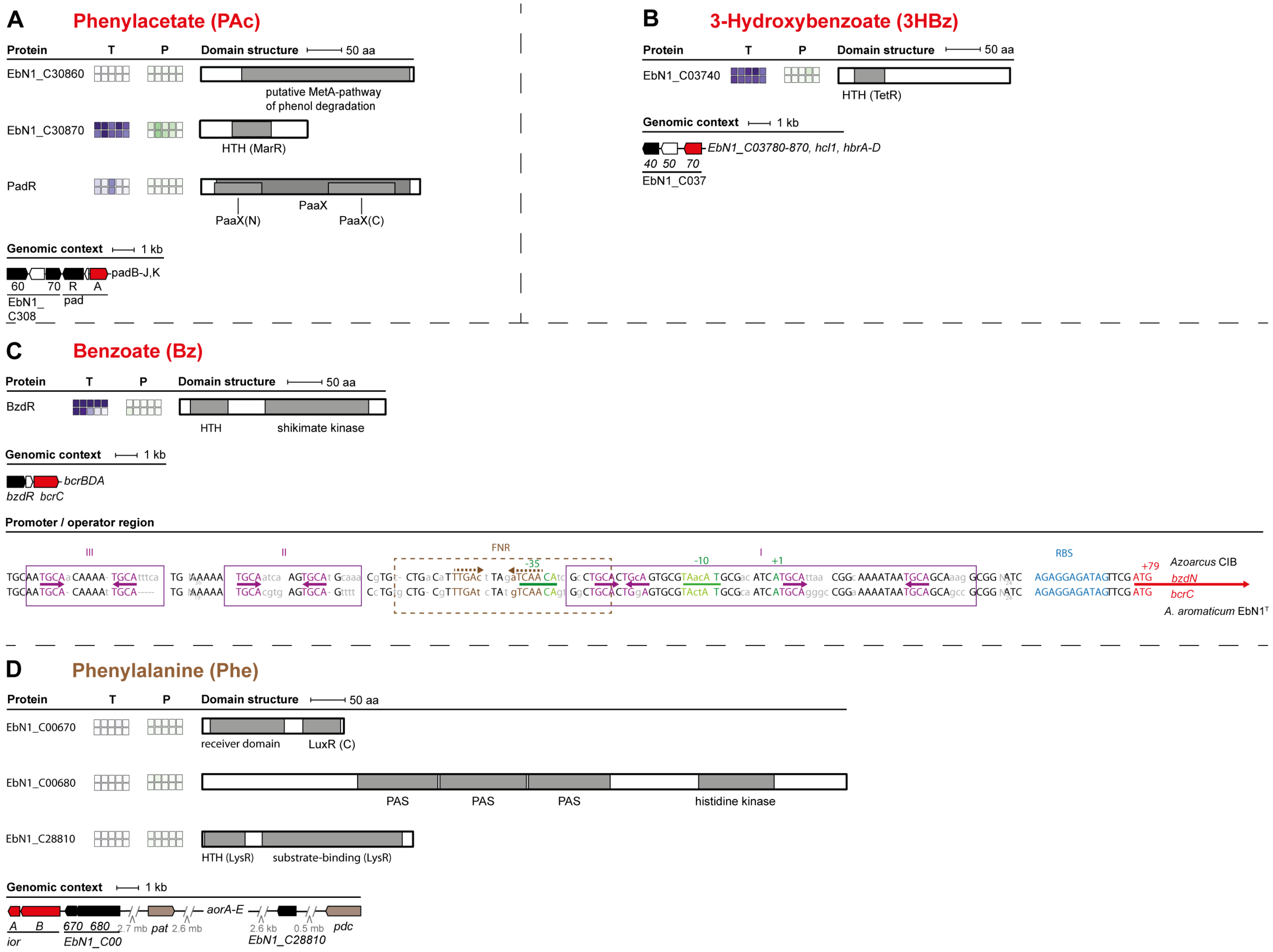

A
Phenylacetate (PAc)
Protein T P Domain structure 50 aa
EbN1_C30860
putative MetA-pathway of phenol degradation
EbN1_C30870
HTH (MarR)
PadR
PaaX
PaaX(N)
PaaX(C)
Genomic context 1 kb
padB-J,K
60 70 R A
EbN1_ C308
pad
B
3-Hydroxybenzoate (3HBz)
Protein T P Domain structure 50 aa
EbN1_C03740
HTH (TetR)
Genomic context 1 kb
EbN1_C03780-870, hcl1, hbrA-D
40 50 70
EbN1_C037
C
Benzoate (Bz)
Protein T P Domain structure 50 aa
BzdR
HTH
shikimate kinase
Genomic context 1 kb
bcrBDA
bzdR bcrC
Promoter / operator region
III
II
FNR
-35
-10
I
+1
RBS
+79
TGCAATGCAaCAAAA- TGCAtttca TG N36 AAAAA TGCAatca AGTGCAt Gcaaa CgTGt- CTGaCaTTTGAct TAgaTCAA CAtc GcCTGCACTGcA GTGCGTAacA TGCGac ATCATGCAttaa CGGcAAAATAATGCAGCAaag GCGG N24 ATC AGAGGAGATAGTTCG ATG
TGCAATGCA- CAAAAt TGCA----- TG N19 AAAAA TGCAcgtg AGTGCA- Gtttt CcTGtg CTG- CgTTTGAt cTAt gTCAA CAgt GgCTGCACTGgAGTGCGTActA TGCGca ATCATGCAgggc CGGaAAAATAATGCAGCAgcc GCGG N24 ATC AGAGGAGATAGTTCG ATG
Azoarcus CIB
bzdN
bcrC
A. aromaticum EbN1T
D
Phenylalanine (Phe)
Protein T P Domain structure 50 aa
EbN1_C00670
receiver domain
LuxR (C)
EbN1_C00680
PAS
PAS
PAS
histidine kinase
EbN1_C28810
HTH (LysR)
substrate-binding (LysR)
Genomic context 1 kb
A B
ior
670 680
EbN1_C00
2.7 mb
pat
2.6 mb
aorA-E
2.6 kb
0.5 mb
EbN1_C28810
pdc

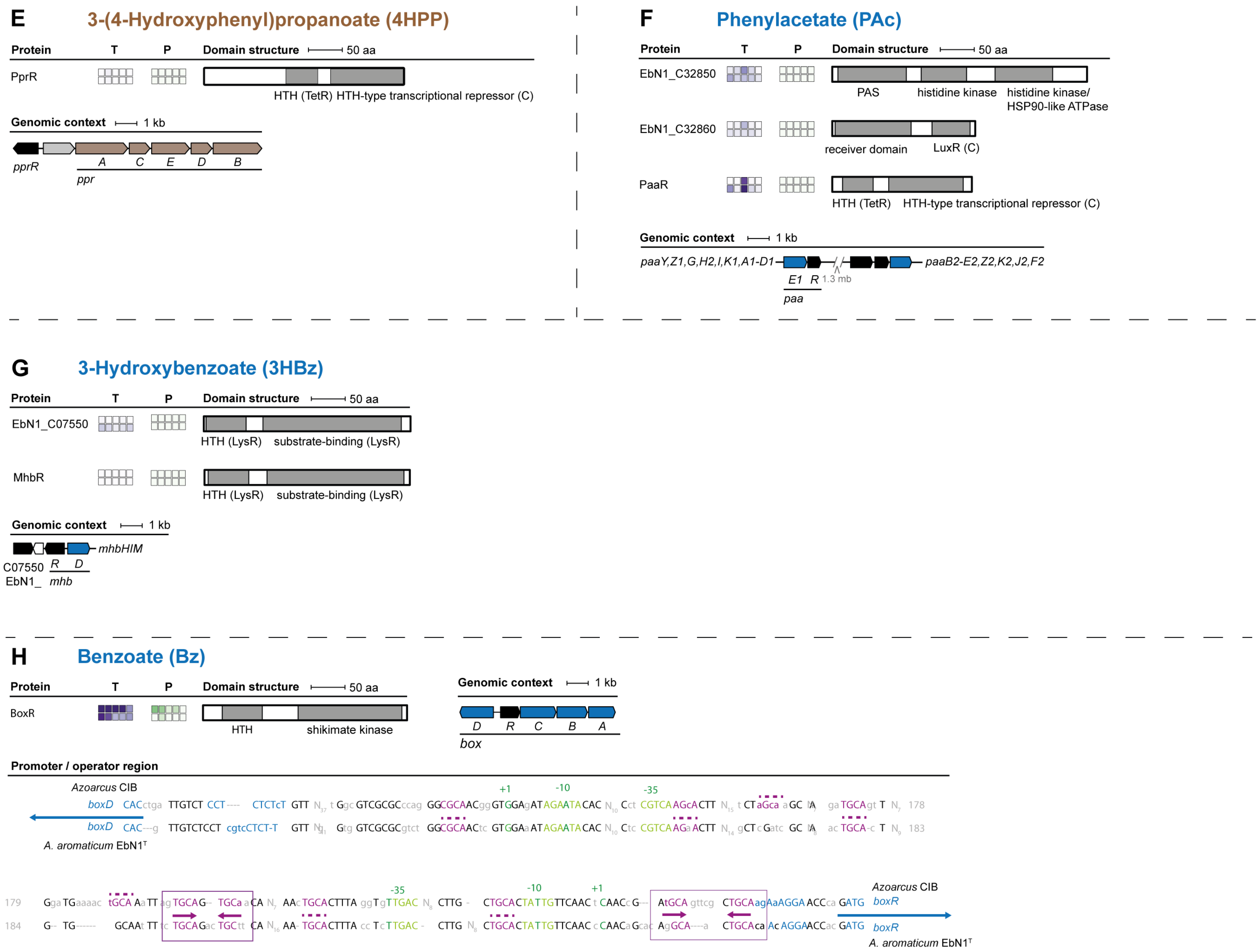
E
3-(4-Hydroxyphenyl)propanoate (4HPP)
Protein
T
P
Domain structure
50 aa
PprR
HTH (TetR)
HTH-type transcriptional repressor (C)
Genomic context
1 kb
pprR
A
C
E
D
B
ppr
F
Phenylacetate (PAc)
Protein
T
P
Domain structure
50 aa
EbN1_C32850
PAS
histidine kinase
histidine kinase/
HSP90-like ATPase
EbN1_C32860
receiver domain
LuxR (C)
PaaR
HTH (TetR)
HTH-type transcriptional repressor (C)
Genomic context
1 kb
paaY,Z1,G,H2,I,K1,A1-D1
E1
R
1.3 mb
paaB2-E2,Z2,K2,J2,F2
paa
G
3-Hydroxybenzoate (3HBz)
Protein
T
P
Domain structure
50 aa
EbN1_C07550
HTH (LysR)
substrate-binding (LysR)
MhbR
HTH (LysR)
substrate-binding (LysR)
Genomic context
1 kb
mhbHIM
C07550
EbN1_
R
D
mhb
H
Benzoate (Bz)
Protein
T
P
Domain structure
50 aa
BoxR
HTH
shikimate kinase
Genomic context
1 kb
D
R
C
B
A
box
Promoter / operator region
Azoarcus CIB
boxD
A. aromaticum EbN1T
+1
-10
-35
178
183
179
184
boxR

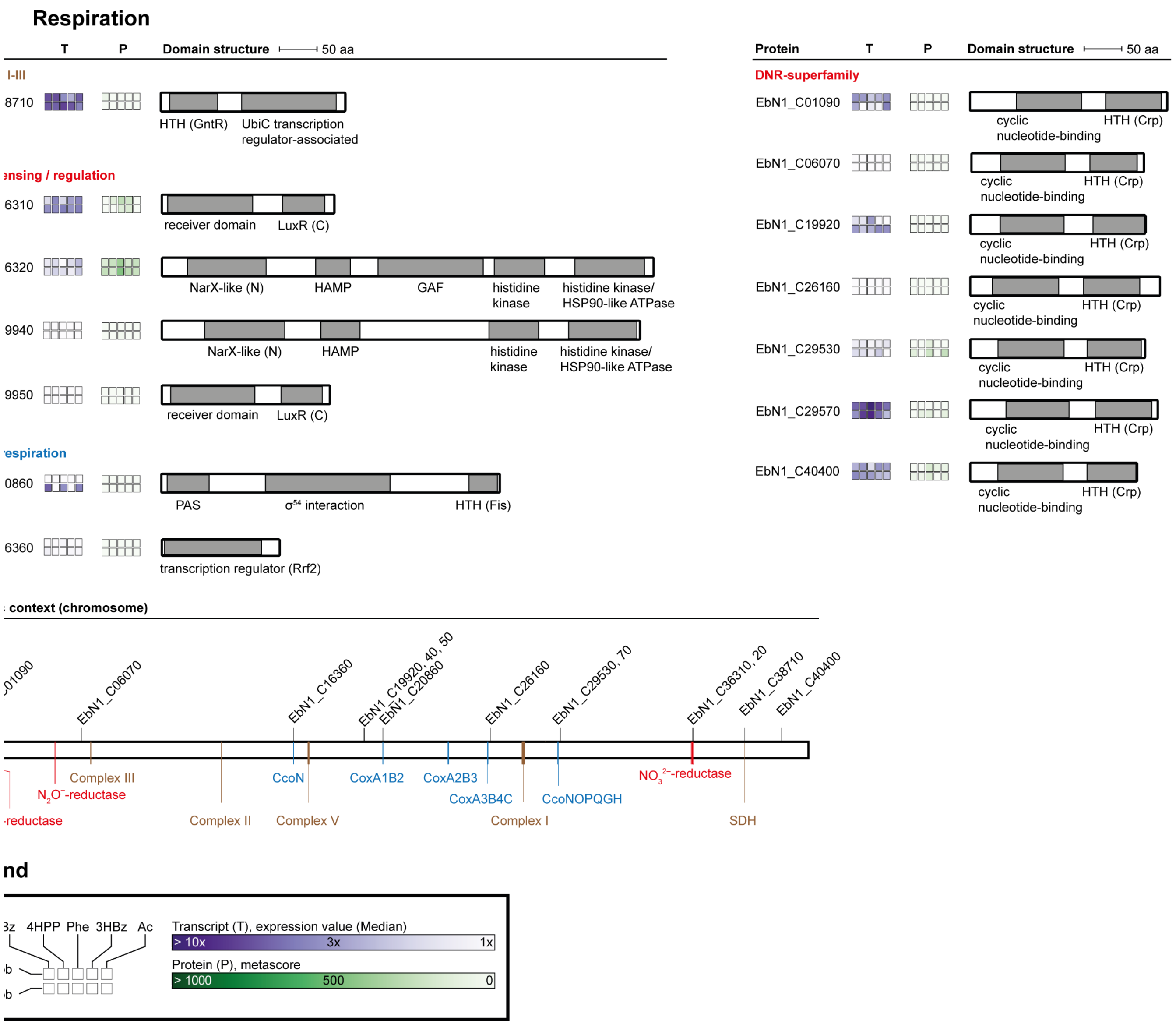

FIG S3 Sensory/regulatory systems potentially involved in transcriptional control of the studied catabolic network. Firstly, the pathway modules involved in anaerobic degradation of (**A**) phenylacetate, (**B**) 3-hydroxy-benzoate, and (**C**) benzoate; in anaerobic & aerobic degradation of (**D**) phenylalanine, and (**E**) 3-(4-hydroxyphenyl) propanoate; and in aerobic degradation of (**F**) phenyl-acetate, (**G**) 3-hydroxybenzoate, and (**H**) benzoate. Secondly, regulators involved in the respiratory network (**I**)
