# Supplementary material for: Systems Biology of Aromatic Compound Catabolism in Facultative Anaerobic Aromatoleum aromaticum EbN1T
Source: mSystems. 2022 Nov 29;7(6):e00685-22. doi: 10.1128/msystems.00685-22 (PMC9765128; doi:10.1128/msystems.00685-22)
Supplement: FIG S4 [file msystems.00685-22-s0004.pdf]

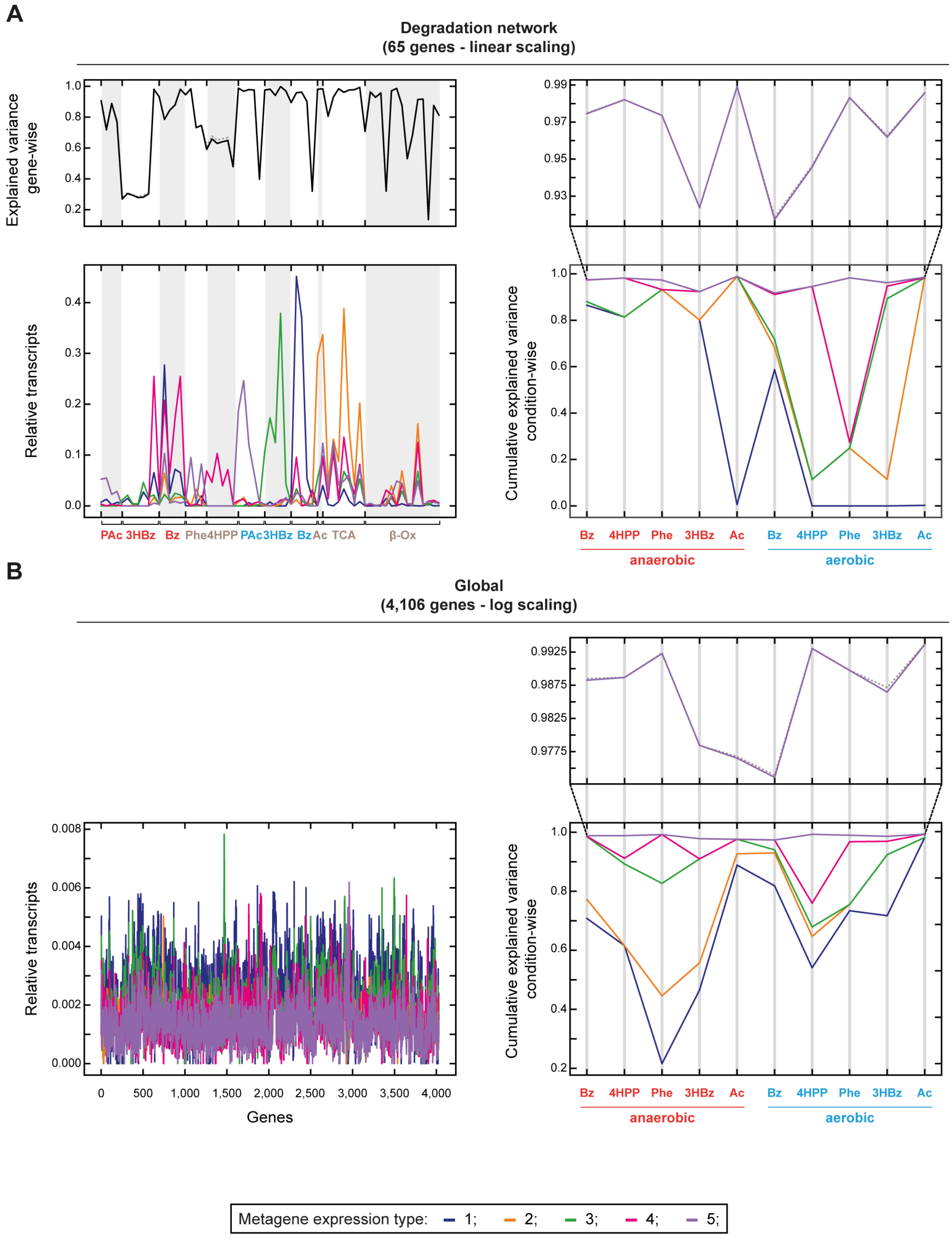
A
Degradation network
(65 genes - linear scaling)
Explained variance gene-wise
Relative transcripts
PAc 3HBz Bz Phe 4HPP PAc 3HBz Bz Ac TCA β-Ox
Cumulative explained variance condition-wise
Bz 4HPP Phe 3HBz Ac Bz 4HPP Phe 3HBz Ac
anaerobic
aerobic
B
Global
(4,106 genes - log scaling)
Relative transcripts
Genes
Cumulative explained variance condition-wise
Bz 4HPP Phe 3HBz Ac Bz 4HPP Phe 3HBz Ac
anaerobic
aerobic
Metagene expression type: 1; 2; 3; 4; 5;

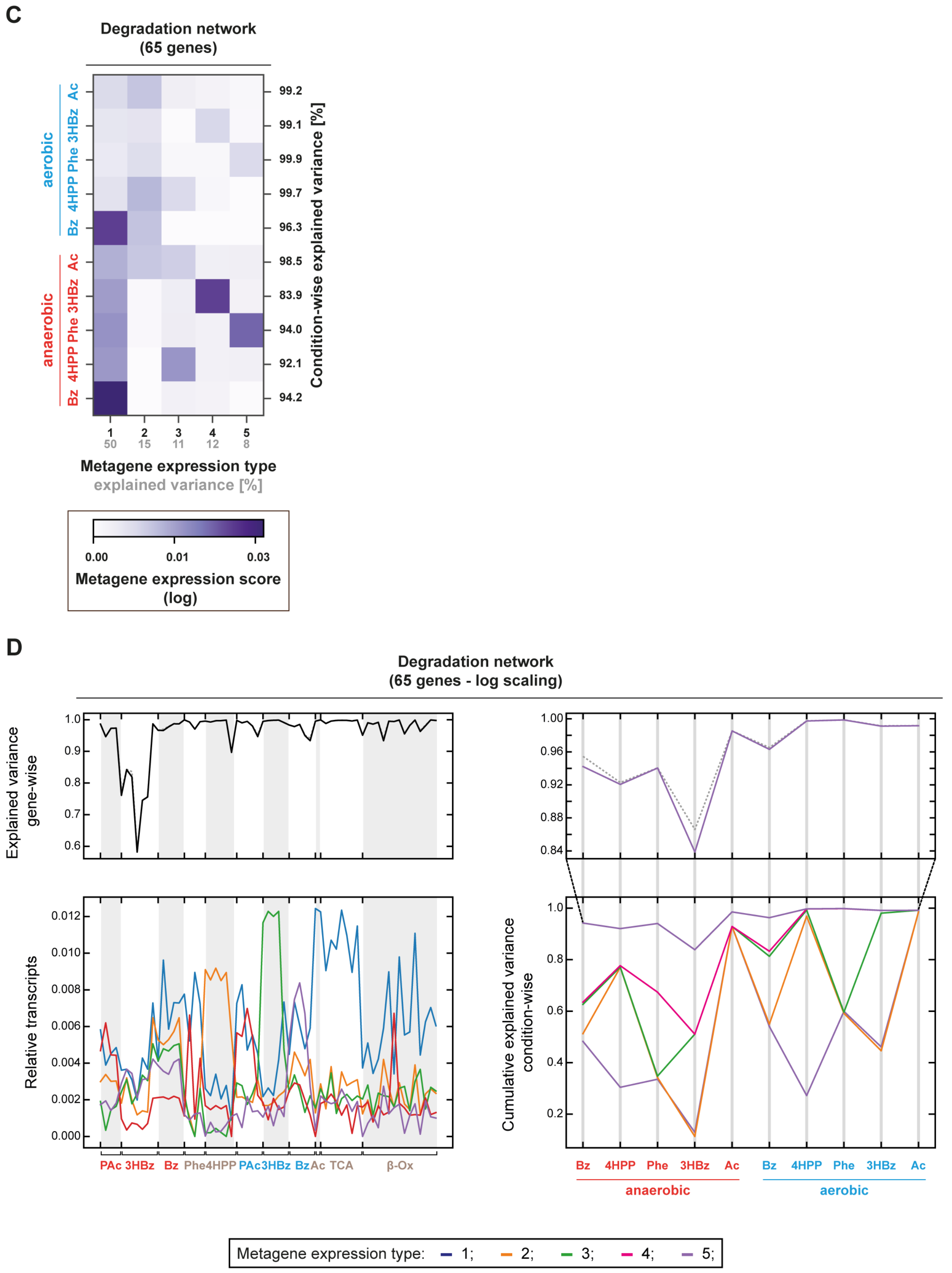

FIG S4 Summary of metagene expression analyses. (**A**) Variance determining factors for the 65 genes in the degradation network according to the model with 5 components whose scores are depicted in Fig. 3A. Top left: Gene-wise explained variance, indicating how much of the original transcriptional variance of a given gene across all growth conditions is explained by the model consisting of 5 metagene expression types. Bottom left: weight matrix W_{ik}, here represented as relative transcript profile across degradation modules (gene index *i* along x-axis) for each metagene expression type (index *k*, colors). Right: Cumulative substrate-wise explained variance for the growth conditions (across all genes), when

adding components (colors) sequentially to the model in ascending order. (**B**) Analysis of all chromosomal genes, with log-transformed transcripts (score matrix shown in Fig. 3B). Left: Relative transcript profile across chromosome (gene index *i* along x-axis) for each metagene expression type (index *k*, colors). Right: Cumulative substrate-wise explained variance. (**C**) Scores matrix for the 65 genes in the degradation network, as in Fig. 5A, but now with log transformed transcripts. (**D**) As in (A), but now with log-transformed transcripts. Note that 5 components here do not suffice to explain more than 90% of substrate-wise variance for 3HBz, anaerobic.
