# Supplementary material for: Systems Biology of Aromatic Compound Catabolism in Facultative Anaerobic Aromatoleum aromaticum EbN1T
Source: mSystems. 2022 Nov 29;7(6):e00685-22. doi: 10.1128/msystems.00685-22 (PMC9765128; doi:10.1128/msystems.00685-22)
Supplement: FIG S1 [file msystems.00685-22-s0001.pdf]

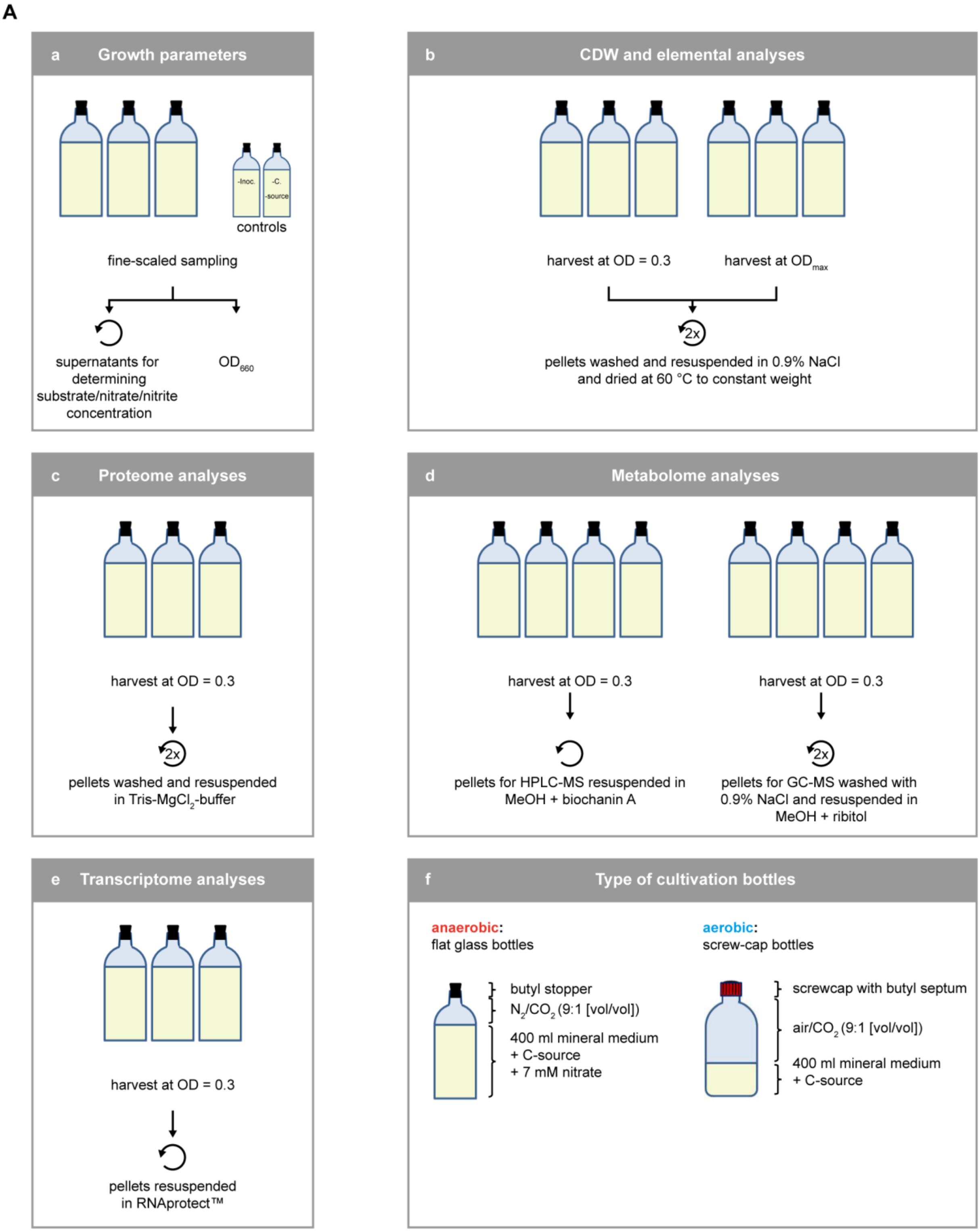
A
a Growth parameters
-Inoc.
-C. -source
controls
fine-scaled sampling
supernatants for determining substrate/nitrate/nitrite concentration
OD660
b CDW and elemental analyses
harvest at OD = 0.3
harvest at ODmax
2x
pellets washed and resuspended in 0.9% NaCl and dried at 60 °C to constant weight
c Proteome analyses
harvest at OD = 0.3
2x
pellets washed and resuspended in Tris-MgCl2-buffer
d Metabolome analyses
harvest at OD = 0.3
harvest at OD = 0.3
pellets for HPLC-MS resuspended in MeOH + biochanin A
2x
pellets for GC-MS washed with 0.9% NaCl and resuspended in MeOH + ribitol
e Transcriptome analyses
harvest at OD = 0.3
pellets resuspended in RNAprotect™
f Type of cultivation bottles
anaerobic:
flat glass bottles
butyl stopper
N2/CO2 (9:1 [vol/vol])
400 ml mineral medium
+ C-source
+ 7 mM nitrate
aerobic:
screw-cap bottles
screwcap with butyl septum
air/CO2 (9:1 [vol/vol])
400 ml mineral medium
+ C-source

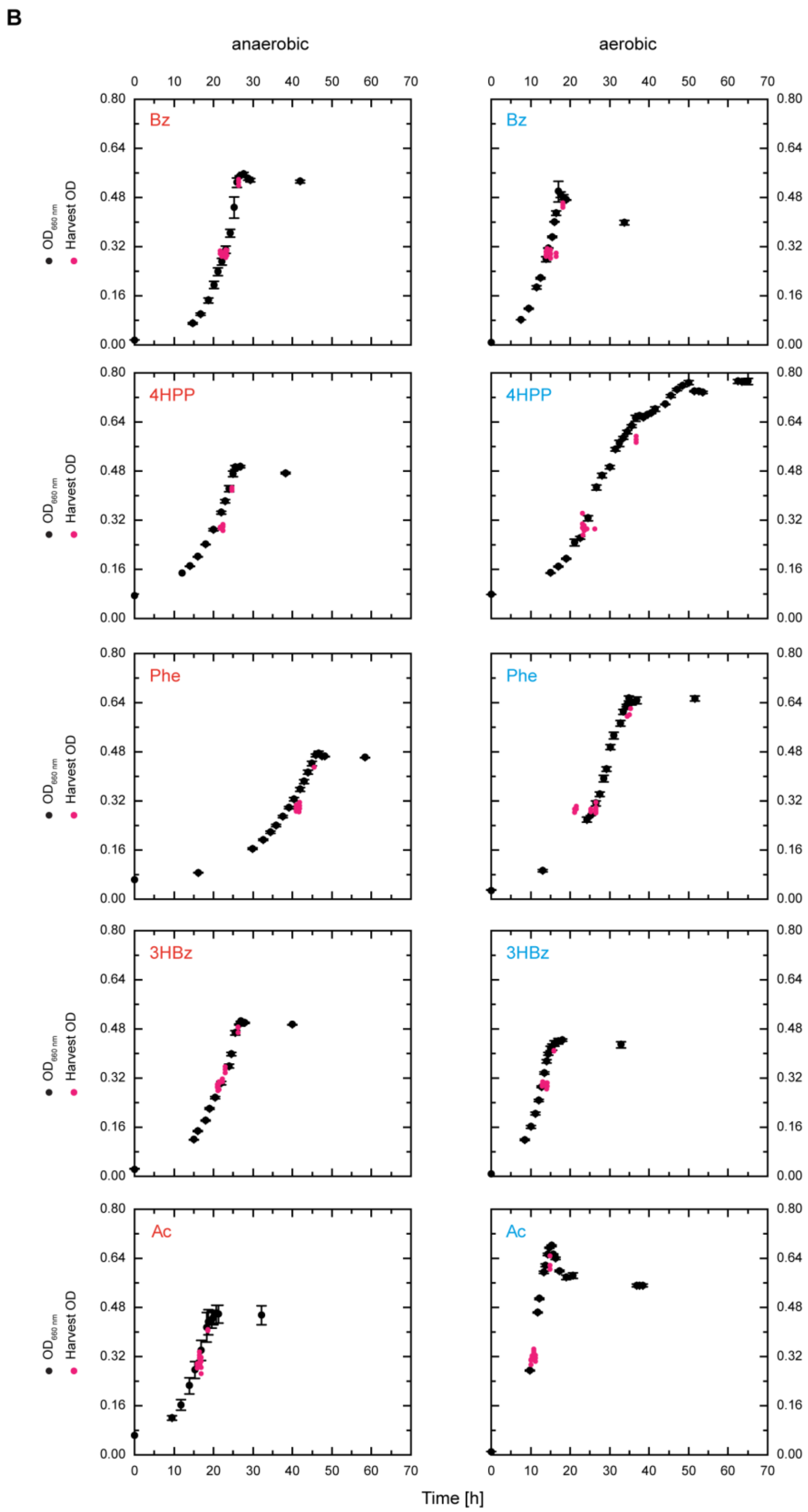
B
anaerobic
aerobic
Bz
4HPP
Phe
3HBz
Ac
● $OD_{660\ nm}$
● Harvest OD
Time [h]

C

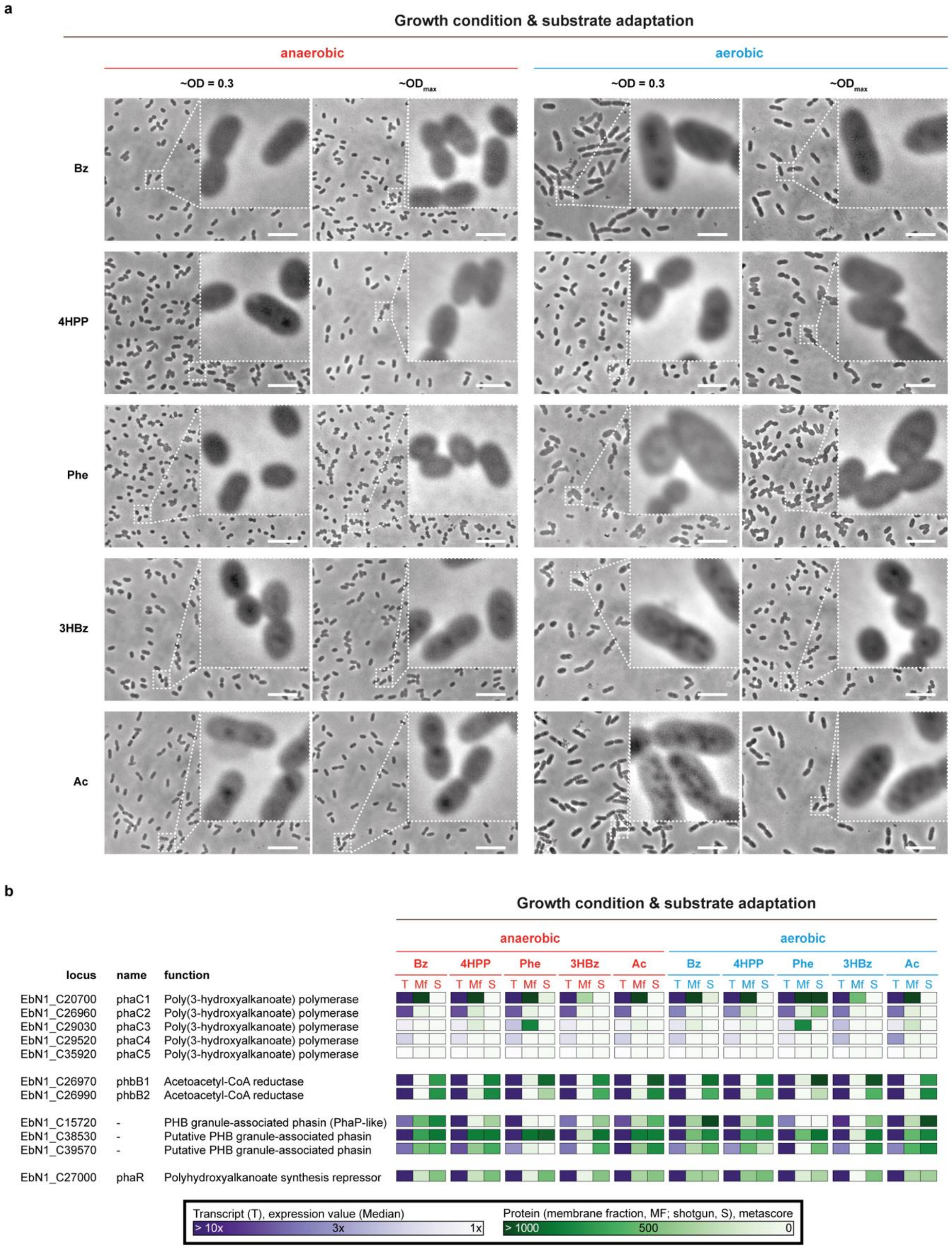
a
Growth condition & substrate adaptation
anaerobic
aerobic
~OD = 0.3
~OD max
Bz
4HPP
Phe
3HBz
Ac
b
Growth condition & substrate adaptation
anaerobic
aerobic
Bz 4HPP Phe 3HBz Ac Bz 4HPP Phe 3HBz Ac
T Mf S
locus name function
EbN1_C20700 phaC1 Poly(3-hydroxyalkanoate) polymerase
EbN1_C26960 phaC2 Poly(3-hydroxyalkanoate) polymerase
EbN1_C29030 phaC3 Poly(3-hydroxyalkanoate) polymerase
EbN1_C29520 phaC4 Poly(3-hydroxyalkanoate) polymerase
EbN1_C35920 phaC5 Poly(3-hydroxyalkanoate) polymerase
EbN1_C26970 phbB1 Acetoacetyl-CoA reductase
EbN1_C26990 phbB2 Acetoacetyl-CoA reductase
EbN1_C15720 - PHB granule-associated phasin (PhaP-like)
EbN1_C38530 - Putative PHB granule-associated phasin
EbN1_C39570 - Putative PHB granule-associated phasin
EbN1_C27000 phaR Polyhydroxyalkanoate synthesis repressor
Transcript (T), expression value (Median)
> 10x 3x 1x
Protein (membrane fraction, MF; shotgun, S), metascore
> 1000 500 0

D

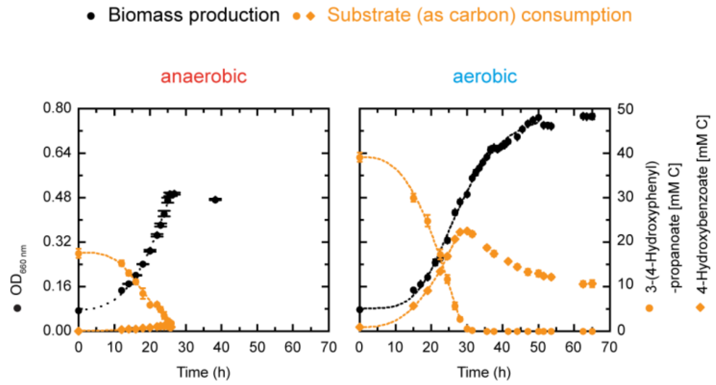

Biomass production
Substrate (as carbon) consumption
anaerobic
aerobic
0.80
0.64
0.48
0.32
0.16
0.00
OD660 nm
0
10
20
30
40
50
60
70
Time (h)
50
40
30
20
10
0
3-(4-Hydroxyphenyl)-propanoate [mM C]
4-Hydroxybenzoate [mM C]

E

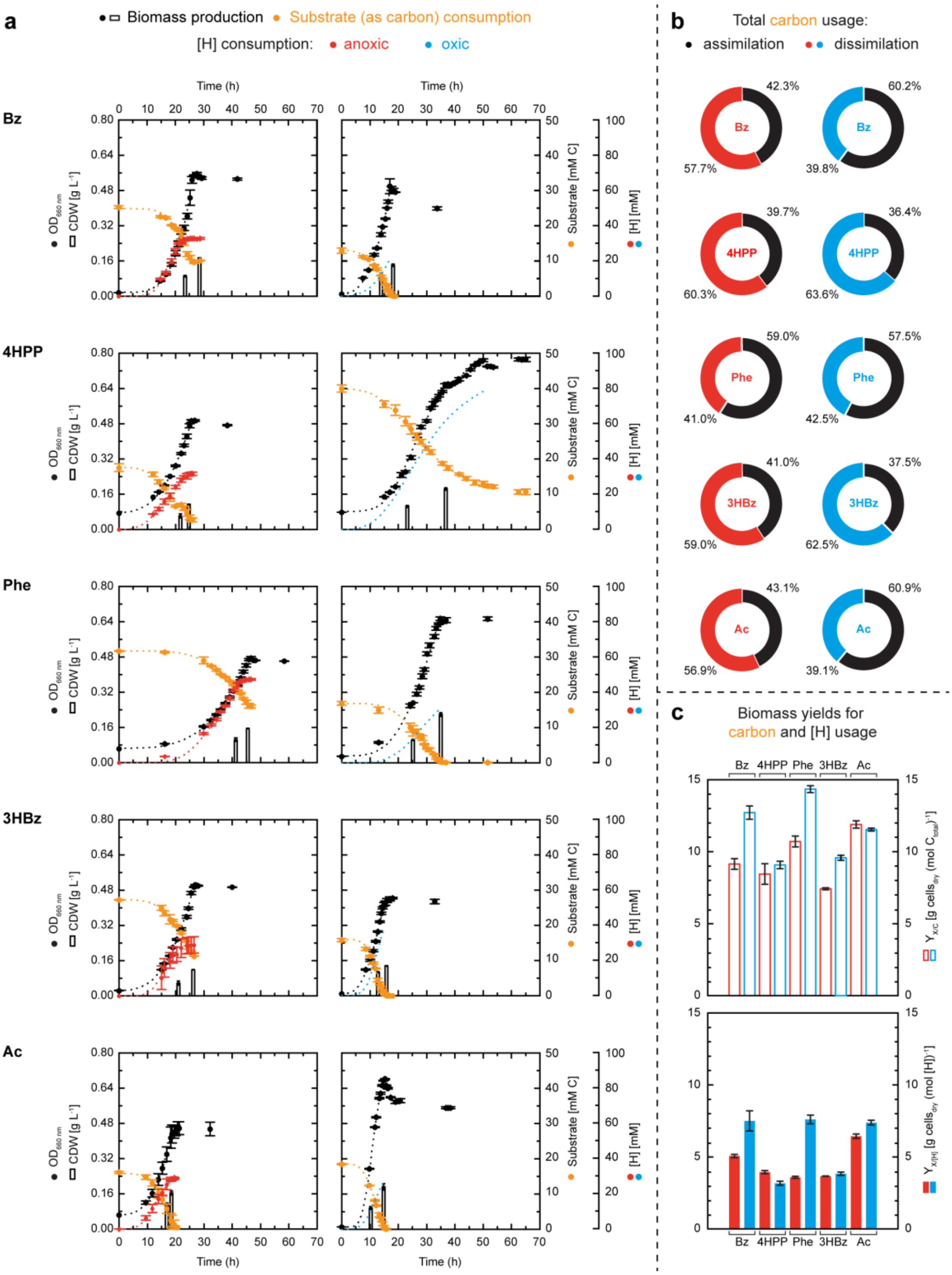
a
Biomass production
Substrate (as carbon) consumption
[H] consumption: anoxic oxic
Time (h)
Bz
4HPP
Phe
3HBz
Ac
OD660 nm
CDW [g L-1]
Substrate [mM C]
[H] [mM]
b
Total carbon usage:
assimilation
dissimilation
42.3%
57.7%
60.2%
39.8%
39.7%
60.3%
36.4%
63.6%
59.0%
41.0%
57.5%
42.5%
41.0%
59.0%
37.5%
62.5%
43.1%
56.9%
60.9%
39.1%
c
Biomass yields for carbon and [H] usage
Bz 4HPP Phe 3HBz Ac
YX/C [g cellsdry (mol Ctotal)-1]
YX/[H] [g cellsdry (mol [H])-1]

F

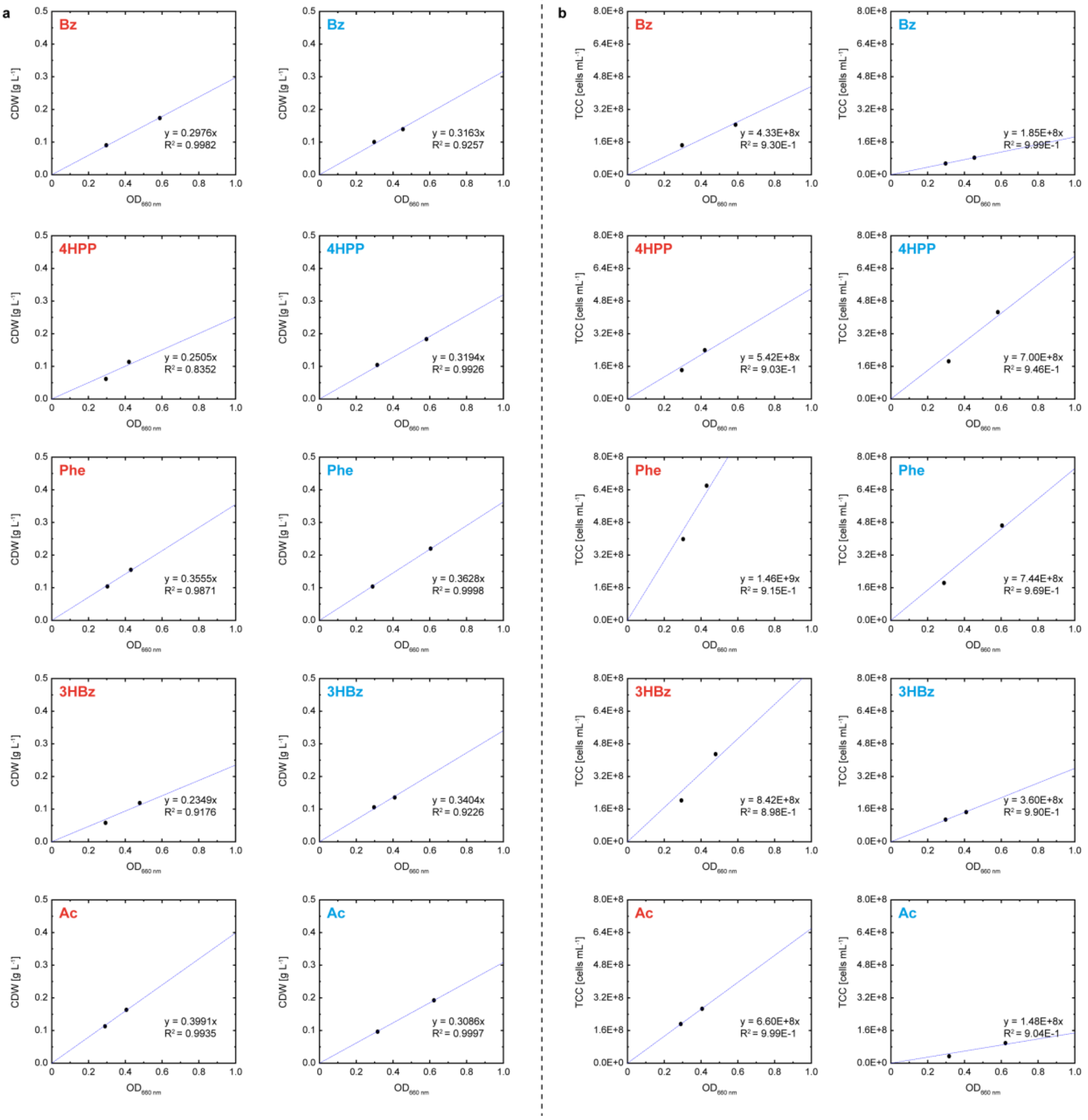

Fig. S1. Physiological data. (**A**) Workflow of the cultivations for differential physiological and multi-OMICS analyses across the ten tested growth conditions. To verify reproducibility of growth for each individual culture, OD660 and concentration of organic substrate as well as nitrate/nitrite (anaerobic cultures only) were determined at beginning of incubation and at time point of harvesting. In total 25 cultures were run in parallel for each tested growth condition. (**B**) Reproducibility of cell harvesting (for multi-OMICS analysis). Coloring: full black dots, reference growth curve; red full dots, harvesting for transcriptomics, proteomics and metabolomics. (**C**) Formation of PHB during anaerobic and aerobic growth of *A. aromaticum* EbN1T provided with either of the five test substrates. (**a**) Phase contrast microscopic images (AxioCamMR, Carl Zeiss AG, Oberkochen, Germany) at ~OD660 = 0.3 (harvesting for multi-OMICS analyses) and ~ODmax; scale bar, 10 µm. (**b**) Genes and proteins possibly involved in PHB formation. Genes are ordered by loci, name and function; expression values of their respective transcripts (T) as well as metascores for their predicted protein products (membrane fraction (Mf) and shotgun (S)) are depicted. (**D**) Growth of *A. aromaticum* EbN1T with 3-(4-hydroxyphenyl)propanoate under anoxic and oxic conditions. Determined growth parameters are based on 3 biological replicates. Consumption of 3-(4-hydroxyphenyl)propanoate and intermediary formation of 4-hydroxybenzoate are displayed as equivalents of carbon. Symbols represent measured

data, while dashed lines represent calculated/simulated data. (**E**) Growth data underlying stoichiometry. (**a**) Course of growth for the ten test conditions integrating biomass production (optical density (OD), cellular dry weight (CDW)), depletion of organic substrate (normalized to mM carbon) and consumption of reducing equivalents [H]. The latter were either calculated from NO_3^- depletion and intermediary NO_2^- formation (anoxic growth) or retro-calculated from [H] derived from dissimilated carbon (oxic growth); results of modelling are indicated by dotted lines. (**b**) Total carbon usage across the ten tested growth conditions according to assimilation and dissimilation. (**c**) Biomass yields based on usage of carbon vs. reducing equivalents [H]. (**F**) Correlation of optical density (OD) with (**a**) cellular dry weight (CDW) and (**b**) total cell count (TCC). The correlations (blue line) are based on the experimental data determined for two growth time points (black full circles). Anoxic vs*.* oxic conditions are indicated by red vs. blue coloring of substrate abbreviations. Correlation factors (y) and degrees of determination of regression (R) are indicated in each subfigure.
