# Supplementary material for: Systems Biology of Aromatic Compound Catabolism in Facultative Anaerobic Aromatoleum aromaticum EbN1T
Source: mSystems. 2022 Nov 29;7(6):e00685-22. doi: 10.1128/msystems.00685-22 (PMC9765128; doi:10.1128/msystems.00685-22)
Supplement: FIG S5 [file msystems.00685-22-s0005.pdf]

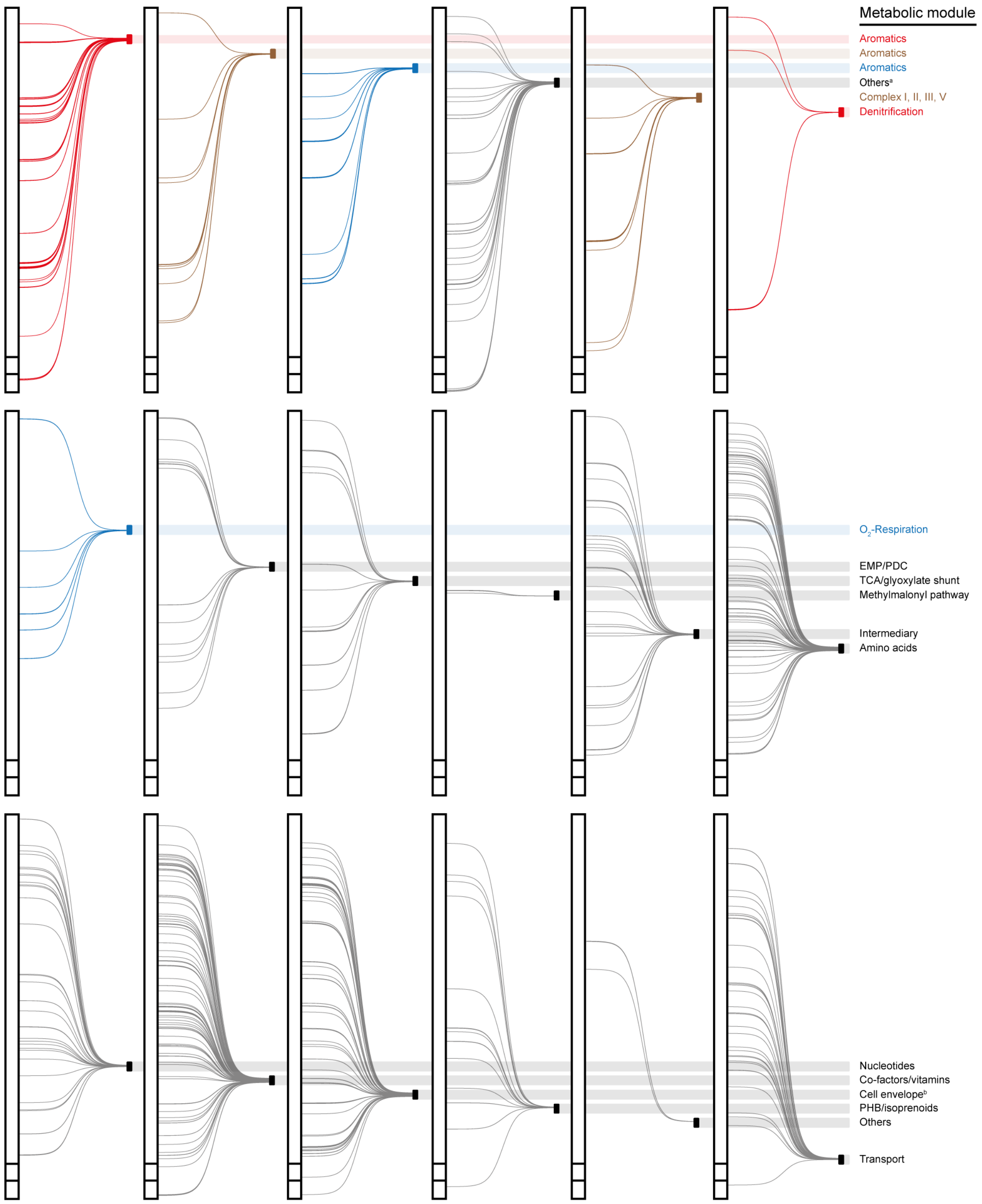

FIG S5 Genomic loci of metabolic modules constituting the metabolic model. Single view per metabolic module as extracted from Fig. 5.
