# Supplementary material for: Systems Biology of Aromatic Compound Catabolism in Facultative Anaerobic Aromatoleum aromaticum EbN1T
Source: mSystems. 2022 Nov 29;7(6):e00685-22. doi: 10.1128/msystems.00685-22 (PMC9765128; doi:10.1128/msystems.00685-22)
Supplement: TABLE S3 [file msystems.00685-22-s0008.pdf]

Experimental and calculated growth rates and calculated C/C yield during the exponential phase for all ten tested conditions.

**Cultivation condition**	**in vivo growth rate [h^{-1}]**	**in silico growth rate [h^{-1}]**	**in silico $Y_{C/C}$ [C C^{-1}]**
Benzoate	0.32	0.28	0.44
Benzoate	0.20	0.21	0.41
3-(4-Hydroxyphenyl)-propanoate	0.10	0.17	0.42
3-(4-Hydroxyphenyl)-propanoate	0.10	0.23	0.44
Phenylalanine	0.13	0.15	0.50
Phenylalanine	0.07	0.09	0.42
3-Hydroxybenzoate	0.37	0.34	0.38
3-Hydroxybenzoate	0.15	0.24	0.41
Acetate	0.58	0.56	0.40
Acetate	0.15	0.15	0.37

Physiological parameters of *A. aromaticum* EbN1^T obtained during anaerobic and aerobic growth.[a]

Growth parameter	Carbon/energy source and electron acceptor [b]									
	Benzoate (Bz) ($C_7H_5O_2$)		3-(4-Hydroxyphenyl)propanoate (4HPP) ($C_9H_9O_3$)		Phenylalanine (Phe) ($C_9H_{10}O_2N$)		3-Hydroxybenzoate (3HBz) ($C_7H_5O_3$)		Acetate (Ac) ($C_2H_3O_2$)	
	anaerobic	aerobic	anaerobic	aerobic	anaerobic	aerobic	anaerobic	aerobic	anaerobic	aerobic
OD_{max}	0.56 ± 0.00	0.51 ± 0.02	0.50 ± 0.00	0.77 ± 0.01	0.48 ± 0.01	0.65 ± 0.01	0.51 ± 0.00	0.44 ± 0.01	0.47 ± 0.01	0.68 ± 0.00
CDW_{max} [g l^{-1}][c]	0.17 ± 0.00	0.16 ± 0.01	0.12 ± 0.00	0.25 ± 0.00	0.17 ± 0.00	0.24 ± 0.00	0.12 ± 0.00	0.15 ± 0.00	0.19 ± 0.00	0.21 ± 0.00
TCC [cells l^{-1}][c]	2.4E11 ± 1.5E09	9.5E10 ± 3.1E09	2.7E11 ± 3.6E08	5.4E11 ± 4.7E09	7.0E11 ± 1.0E10	4.9E11 ± 6.3E09	4.3E11 ± 7.5E08	1.6E11 ± 1.8E09	3.1E11 ± 7.8E09	1.0E11 ± 3.3E08
Biomass elemental composition	$<C_4 H_{6.5} O_{1.2} N_{0.9}>$	$<C_4 H_{7.1} O_{1.3} N_{0.8}>$	$<C_4 H_{7.1} O_{1.2} N_{0.9}>$	$<C_4 H_{7.2} O_{1.7} N_{0.9}>$	$<C_4 H_{6.9} O_{0.6} N_{0.9}>$	$<C_4 H_{7.2} O_{1.6} N_{1.0}>$	$<C_4 H_{6.9} O_{1.1} N_{0.8}>$	$<C_4 H_{7.7} O_{2.2} N_{1.0}>$	$<C_4 H_{6.7} O_{2.5} N_{0.8}>$	$<C_4 H_{6.6} O_{1.3} N_{0.7}>$
C_{total} consumption [mM]	18.17 ± 0.83	12.83 ± 0.78	14.85 ± 1.17	27.03 ± 0.68	15.81 ± 0.47	16.54 ± 0.36	15.99 ± 0.12	15.81 ± 0.47	15.76 ± 0.36	18.22 ± 0.17
[H] consumption [mM]	32.79 ± 0.55[e]	21.98 ± 2.48[d]	31.06 ± 0.84[e]	77.66 ± 3.28[d]	47.24 ± 0.46[e]	31.27 ± 1.37[d]	32.41 ± 0.17[e]	39.56 ± 1.63[d]	29.12 ± 0.14[e]	28.48 ± 0.66[d]
C demand [mmol C_{total} (g cells$_{dry}$)$^{-1}$]	109.51 ± 4.34	78.77 ± 2.79	119.30 ± 9.55	110.21 ± 3.18	93.54 ± 3.37	69.70 ± 1.15	134.72 ± 1.15	104.52 ± 1.92	84.11 ± 1.75	86.69 ± 0.76
[H] demand [mmol [H] (g cells$_{dry}$)$^{-1}$][e]	197.69 ± 4.59	134.86 ± 11.95	253.96 ± 7.06	316.63 ± 14.51	279.49 ± 5.24	131.75 ± 5.09	273.23 ± 0.90	261.39 ± 7.68	155.44 ± 3.76	135.48 ± 3.06
C_{diss} [%][f]	57.73 ± 1.72	39.84 ± 2.17	60.28 ± 3.37	63.61 ± 1.03	41.01 ± 2.08	42.51 ± 0.96	58.98 ± 0.35	62.51 ± 0.68	56.92 ± 0.91	39.07 ± 0.54
μ_{max} [h^{-1}]	0.20 ± 0.00	0.32 ± 0.00	0.10 ± 0.00	0.10 ± 0.00	0.07 ± 0.00	0.13 ± 0.01	0.15 ± 0.00	0.37 ± 0.02	0.15 ± 0.00	0.58 ± 0.01
t_D [h][g]	3.49 ± 0.03	2.15 ± 0.01	7.27 ± 0.09	6.61 ± 0.02	10.55 ± 0.05	5.28 ± 0.23	4.57 ± 0.02	1.86 ± 0.10	4.75 ± 0.15	1.20 ± 0.02
$q_{X/X}$ [h^{-1}][h]	0.20 ± 0.01	0.32 ± 0.02	0.10 ± 0.00	0.10 ± 0.01	0.07 ± 0.00	0.13 ± 0.01	0.15 ± 0.00	0.39 ± 0.03	0.15 ± 0.00	0.60 ± 0.05
$q_{C/X}$ [mmol C_{total} (g cells$_{dry}$)$^{-1}$h^{-1}][h]	18.54 ± 0.83	23.60 ± 0.77	16.44 ± 3.59	14.81 ± 0.98	7.53 ± 0.61	10.72 ± 0.63	21.27 ± 0.54	32.83 ± 1.44	14.80 ± 0.85	51.31 ± 6.41
$q_{[H]/X}$ [mmol [H] (g cells$_{dry}$)$^{-1}$h^{-1}][h]	135.69 ± 13.03	40.72 ± 0.90	43.72 ± 6.10	41.87 ± 2.77	35.34 ± 1.73	20.25 ± 1.19	91.88 ± 9.88	82.09 ± 3.61	43.71 ± 1.49	79.23 ± 10.96

[a] All values are based on 3 biological replicates.
[b] All substrates were provided as sole source of carbon and energy in defined, mineral medium. Initial substrate concentrations amounted for approximately 2/4 mM for Bz, Phe and 3HBz, 4/2 mM for 4HPP and 8/8 mM for Ac. Anoxic media was initially supplemented with 7 mM or 10 mM nitrate for Bz, 4HPP, 3HBz, Ac or Phe, respectively.
[c] Calculated values using correlation of OD_{660} and CDW/Cell values (see Fig.S7).
[d] Based on calculated values of [H] released from dissimilated carbon.
[e] Based on measurement of nitrate and nitrite consumption.
[f] Carbon dissimilated (C_{diss}), based on calculated values of assimilated carbon using the assimilation equation (see Tab. S12) and CDW_{max}-values.
[g] Doubling time.
[h] Averaged maximal biomass specific rates for growth ($q_{X/X}$), carbon ($q_{C/X}$) and reducing equivalents ($q_{[H]/X}$).

Stoichiometric equations for assimilation, dissimilation and metabolism for all ten tested conditions in *A. aromaticum* EbN1^T. The substrate-specific elemental composition (<CHON>) of biomass was determined at OD_{max} in biological triplicates.

Carbon and energy source	Equations (at OD_{max})
Benzoate	
Assimilation equation	$C_7H_5O_2^- + 1.15\ H_2O + 1.39\ NH_4^+ \rightarrow 1.68 < C_4H_{7.1}O_{1.3}N_{0.8} > + 0.30\ HCO_3^- + 0.68\ H^+$
Dissimilation equation	$C_7H_5O_2^- + 4.00\ H_2O + 7.50\ O_2 \rightarrow\ 7.00\ HCO_3^- + 6.00\ H^+$
Metabolic equation	$C_7H_5O_2^- + 2.58\ H_2O + 3.75\ O_2 + 0.69\ NH_4^+ \rightarrow 0.84\ < C_4H_{7.1}O_{1.3}N_{0.8} > + 3.65\ HCO_3^- + 3.34\ H^+$
Benzoate	
Assimilation equation	$C_7H_5O_2^- + 0.44\ H_2O + 1.51\ NH_4^+ \rightarrow 1.72 < C_4H_{6.5}O_{1.2}N_{0.9} > + 0.14\ HCO_3^- + 0.65\ H^+$
Dissimilation equation	$C_7H_5O_2^- + 1.00\ H_2O + 6.00\ NO_3^- \rightarrow\ 7.00\ HCO_3^- + 3.00\ N_2$
Metabolic equation	$C_7H_5O_2^- + 0.72\ H_2O + 3.00\ NO_3^- + 0.76\ NH_4^+ \rightarrow 0.86\ < C_4H_{6.5}O_{1.2}N_{0.9} > + 3.57\ HCO_3^- + 0.32\ H^+ + 1.50\ N_2$
3-(4-Hydroxyphenyl)-propanoate	
Assimilation equation	$C_9H_9O_3^- +\ 0.43\ HCO_3^- + 2.23\ NH_4^+ \rightarrow 2.36 < C_4H_{7.2}O_{1.7}N_{0.9} > +0.80\ H^+ + 0.33\ H_2O$
Dissimilation equation	$C_9H_9O_3^- + 4.00\ H_2O + 10.00\ O_2 \rightarrow\ 9.00\ HCO_3^- + 8.00\ H^+$
Metabolic equation	$C_9H_9O_3^- + 1.84\ H_2O + 5.00\ O_2 + 1.11\ NH_4^+ \rightarrow 1.18\ < C_4H_{7.2}O_{1.7}N_{0.9} > + 4.29\ HCO_3^- + 4.40\ H^+$
3-(4-Hydroxyphenyl)-propanoate	
Assimilation equation	$C_9H_9O_3^- + 0.02\ H_2O + 1.96\ NH_4^+ \rightarrow 2.22 < C_4H_{7.1}O_{1.2}N_{0.9} > + 0.14\ HCO_3^- + 1.09\ H^+$
Dissimilation equation	$C_9H_9O_3^- + 8.00\ NO_3^- \rightarrow\ 9.00\ HCO_3^- + 4.00\ N_2$
Metabolic equation	$C_9H_9O_3^- + 0.01\ H_2O + 4.00\ NO_3^- + 0.98\ NH_4^+ \rightarrow 1.11\ < C_4H_{7.1}O_{1.2}N_{0.9} > + 4.57\ HCO_3^- + 0.55\ H^+ + 2.00\ N_2$
Phenylalanine	
Assimilation equation	$C_9H_{10}O_2N^- +\ 0.45\ HCO_3^- + 0.37\ H_2O + 1.47\ NH_4^+ \rightarrow 2.36 < C_4H_{7.2}O_{1.6}N_{1.0} > +0.02\ H^+$
Dissimilation equation	$C_9H_{10}O_2N^- + 5.00\ H_2O + 10.00\ O_2 \rightarrow\ 9.00\ HCO_3^- + 7.00\ H^+ + 1.00\ NH_4^+$
Metabolic equation	$C_9H_{10}O_2N^- + 2.69\ H_2O + 5.00\ O_2 + 0.23\ NH_4^+ \rightarrow 1.18\ < C_4H_{7.2}O_{1.6}N_{1.0} > + 4.28\ HCO_3^- + 3.51\ H^+$
Phenylalanine	
Assimilation equation	$C_9H_{10}O_2N^- + 0.87\ H_2O + 0.96\ NH_4^+ \rightarrow 2.11 < C_4H_{6.9}O_{0.6}N_{0.9} > + 0.55\ HCO_3^- + 0.51\ H^+$
Dissimilation equation	$C_9H_{10}O_2N^- + 1.00\ H^+ + 1.00\ H_2O + 8.00\ NO_3^- \rightarrow\ 9.00\ HCO_3^- + 4.00\ N_2 + 1.00\ NH_4^+$
Metabolic equation	$C_9H_{10}O_2N^- + 0.25\ H^+ + 0.94\ H_2O + 4.00\ NO_3^- \rightarrow 1.06\ < C_4H_{6.9}O_{0.6}N_{0.9} > + 4.77\ HCO_3^- + 2.00\ N_2 + 0.02\ NH_4^+$
3-Hydroxybenzoate	
Assimilation equation	$C_7H_5O_3^- + 1.18\ H_2O + 1.70\ NH_4^+ \rightarrow 1.71 < C_4H_{7.7}O_{2.2}N_{1.0} > + 0.16\ HCO_3^- + 0.86\ H^+$
Dissimilation equation	$C_7H_5O_3^- + 4.00\ H_2O + 7.00\ O_2 \rightarrow\ 7.00\ HCO_3^- + 6.00\ H^+$
Metabolic equation	$C_7H_5O_3^- + 2.59\ H_2O + 3.50\ O_2 + 0.85\ NH_4^+ \rightarrow 0.86\ < C_4H_{7.7}O_{2.2}N_{1.0} > +3.58\ HCO_3^- + 3.43\ H^+$
3-Hydroxybenzoate	
Assimilation equation	$C_7H_5O_3^- + 1.33\ H_2O + 1.23\ NH_4^+ \rightarrow 1.52 < C_4H_{6.9}O_{1.1}N_{0.8} > + 0.91\ HCO_3^- + 1.14\ H^+$
Dissimilation equation	$C_7H_5O_3^- + 1.20\ H_2O + 5.60\ NO_3^- \rightarrow\ 7.00\ HCO_3^- + 0.40\ H^+ + 2.80\ N_2$
Metabolic equation	$C_7H_5O_3^- + 1.27\ H_2O + 2.80\ NO_3^- + 0.61\ NH_4^+ \rightarrow 0.76\ < C_4H_{6.9}O_{1.1}N_{0.8} > + 3.95\ HCO_3^- + 0.77\ H^+ + 1.40\ N_2$
Acetate	
Assimilation equation	$C_2H_3O_2^- + 0.48\ H^+ + 0.30\ NH_4^+ \rightarrow 0.44 < C_4H_{6.6}O_{1.3}N_{0.7} > + 0.22\ HCO_3^- + 0.75\ H_2O$
Dissimilation equation	$C_2H_3O_2^- + 2.00\ O_2 \rightarrow\ 2.00\ HCO_3^- + 1.00\ H^+$
Metabolic equation	$C_2H_3O_2^- + 1.00\ O_2 + 0.15\ NH_4^+ \rightarrow 0.22\ < C_4H_{6.6}O_{1.3}N_{0.7} > +1.11\ HCO_3^- + 0.26\ H^+ + 0.38\ H_2O$
Acetate	
Assimilation equation	$C_2H_3O_2^- +\ 0.09\ HCO_3^- + 0.66\ H^+ + 0.43\ NH_4^+ \rightarrow 0.52 < C_4H_{6.7}O_{2.5}N_{0.8} > +1.00\ H_2O$
Dissimilation equation	$C_2H_3O_2^- + 0.60\ H^+ + 1.60\ NO_3^- \rightarrow\ 2.00\ HCO_3^- + 0.80\ H_2O + 0.80\ N_2$
Metabolic equation	$C_2H_3O_2^- + 0.63\ H^+ + 0.80\ NO_3^- + 0.22\ NH_4^+ \rightarrow 0.26\ < C_4H_{6.7}O_{2.5}N_{0.8} > + 0.95\ HCO_3^- + 0.90\ H_2O + 0.40\ N_2$
