# Supplementary material for: Systems Biology of Aromatic Compound Catabolism in Facultative Anaerobic Aromatoleum aromaticum EbN1T
Source: mSystems. 2022 Nov 29;7(6):e00685-22. doi: 10.1128/msystems.00685-22 (PMC9765128; doi:10.1128/msystems.00685-22)
Supplement: TABLE S4 [file msystems.00685-22-s0009.pdf]

TABLE S4. Enzymatic reactions implemented in metabolic model.

Metabolic reactions	in all respective simulations	in at least one	exclusively
Overall active reactions	344	429	-
In acetate cultures	356	376	0
In aromatic compounds	344	429	73
In aerobic cultures	357	402	28
In anaerobic cultures	353	401	27
In aerobic aromates	357	401	28
in anaerobic aromates	358	401	28
